# Cytoplasmic Shift of Interferon Regulatory Factors Co‐Evolved With Jawed Vertebrate Innate Immunity

**DOI:** 10.1002/jmv.70247

**Published:** 2025-02-20

**Authors:** Vanessa Hubing, Avery Marquis, Chanasei Ziemann, Hideaki Moriyama, Etsuko N. Moriyama, Luwen Zhang

**Affiliations:** ^1^ School of Biological Sciences University of Nebraska Lincoln Nebraska USA; ^2^ Center for Plant Science Innovation University of Nebraska Lincoln Nebraska USA; ^3^ Nebraska Center for Virology University of Nebraska Lincoln Nebraska USA

**Keywords:** evolution, inflammation, innate immunity, interferon, IRF, jaw

## Abstract

The emergence of jaws in early vertebrates introduced a novel feeding apparatus and powerful oral defenses, but it also increased the risk of physical injury and pathogen exposure. Interferon regulatory factors (IRFs) play critical roles in orchestrating innate immunity and inflammation in response to invading microbes and tissue damage, with their subcellular localization being essential to some IRFs' function. Our results indicate that IRF members underwent independent expansion and diversification in two distinct vertebrate lineages: jawed and jawless vertebrates. The jawed vertebrate‐specific factor, IRF5, has maintained conserved nuclear export sequences throughout evolution, while newly diversified IRF members in jawed vertebrates have acquired cytoplasmic localization. This cytoplasmic shift particularly affected IRFs involved in type I interferon (IFN) signaling (IRF3, IRF5, IRF7, and IRF9), suggesting co‐evolution with the development of the type I IFN system in jawed animals. Interestingly, although IRF9 is inherently nuclear, its association with Signal Transducer and Activator of Transcription 2 (STAT2) has led to its cytoplasmic localization. Additionally, IRF6, another jawed vertebrate‐specific factor, plays a crucial role in jaw development, reflecting an evolutionary adaptation that aligns structural innovations with immune function. Our findings suggest that the evolution of jaws coincided with the adoption of cytoplasmic localization in IRF members, potentially enhancing rapid immune responses to meet the immunological challenges posed by the predatory lifestyle of early jawed vertebrates.

## 1 Introduction

The evolution of jaws marked a transformative innovation in early vertebrates, fundamentally altering their interaction with the environment. This transition from filter feeding to active predation not only enabled access to new food resources but also introduced novel immunological challenges: powerful jaws increased risks of physical injury while consuming larger prey exposed early jawed vertebrates to more diverse pathogen communities. These new environmental pressures likely drove the evolution of more sophisticated immune defenses. The innate immune system and inflammation, constituting the first line of defense against pathogens, injury, and stress, play crucial roles in both immediate protection and broader physiological functions, suggesting their potential co‐evolution with jaw‐related innovations.

Interferon regulatory factors (IRFs), a family of transcription factors, are key orchestrators of innate immune and inflammatory responses. These transcription factors share a conserved DNA‐binding domain (DBD) in their N‐terminus that recognizes specific DNA sequences [1]. The IRF‐association domain (IAD) at the C‐terminus determines specific biological functions (Supporting Information S1: Figure S1). A distinctive feature of several IRF family members, particularly those involved in interferon (IFN) signaling (IRF3, 5, 7, and 9), is their cytoplasmic localization in unstimulated cells. This spatial regulation through nuclear‐cytoplasmic shuttling serves as a crucial control mechanism for their function [2, 3, 4, 5, 6].

Among these cytoplasmic IRFs, IRF5 exemplifies the importance of spatial regulation in immune function. Expressed in immune cells including macrophages and dendritic cells, IRF5 responds to pattern recognition receptor activation by translocating from its cytoplasmic location to the nucleus, where it induces pro‐inflammatory cytokines and type I IFNs [7, 8]. Through this regulated trafficking, IRF5 influences both innate and adaptive immunity, and its dysregulation is linked to autoimmune conditions such as systemic lupus erythematosus [9, 10].

While significant advances have been made in understanding IRF biology, the evolution of their nuclear‐cytoplasmic trafficking mechanisms remains poorly characterized [11, 12, 13, 14, 15]. Here, we conducted phylogenetic and sequence analyses of IRF proteins focusing especially on the transition from jawless to jawed vertebrates. Our findings revealed that this jawless‐to‐jawed transition was accompanied by both lineage‐specific expansion of the IRF family and acquisition of cytoplasmic localization by key IRFs, suggesting an evolutionary innovation that enhanced immune response regulation through spatial control of these transcription factors.

## 2 Results

### 2.1 Independent Expansion and Evolution of IRF Family Members in Jawless and Jawed Vertebrates

**Figure 1:**
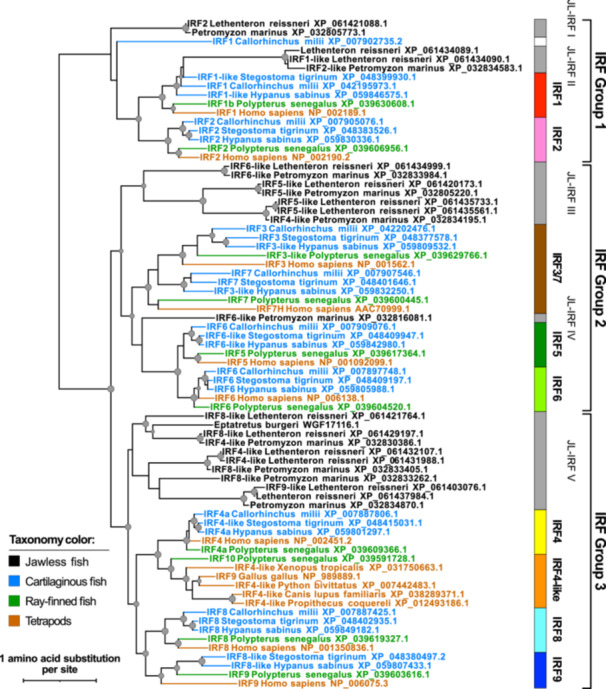
Phylogenetic relationships among IRF protein families. The maximum likelihood phylogeny was reconstructed using the IRF protein sequences from representative jawless and jawed vertebrate species. The internal nodes supported by 80% or higher by both ultrafast bootstrap and SH‐aLRT branch tests are denoted by gray dots with the size correlated with the ultrafast bootstrap values (80%−100%). Terminal branches and sequence names are colored based on the taxonomic groups as shown in the color legend. The information of the sequences used is listed in Supporting Information S1: Table S1.

To understand the evolutionary dynamics of the IRF family, particularly in relation to jaw evolution, we conducted a phylogenetic analysis incorporating IRF proteins from multiple lineages of both jawless and jawed vertebrates. Consistent with previous studies [11, 12, 13, 14, 16], vertebrate IRF proteins cluster into three major groups (Groups 1−3 in Figure 1; corresponding to “IRF1‐G,” “IRF3‐G” and “IRF‐7G,” and “IRF4‐G” in a previous report [12]). While these three major groups are present in both jawless and jawed vertebrates, our analysis further revealed independent expansion in each of these lineages after their divergence. In jawed vertebrates, Group 1 expanded to include the IRF2 subfamily, while Groups 2 and 3 gave rise to IRF3/7 and IRF5/6 subfamilies (Group 2), and IRF4, 8, and 9 subfamilies (Group 3). In contrast, in jawless vertebrates, the IRF family underwent independent expansion resulting in unique subfamilies: JL‐IRF III (Group 2) and JL‐IRF V (Group 3) (Figure 1). This pattern of independent subfamily expansion suggests that IRF diversification may be linked to the evolution of lineage‐specific features, including jaws and their associated immune functions in jawed vertebrates.

### 2.2 Conservation of Nuclear Export Signal (NES) and Serine‐Rich Region (SRR) in Jawed Vertebrate IRF5 Proteins

**Figure 2:**
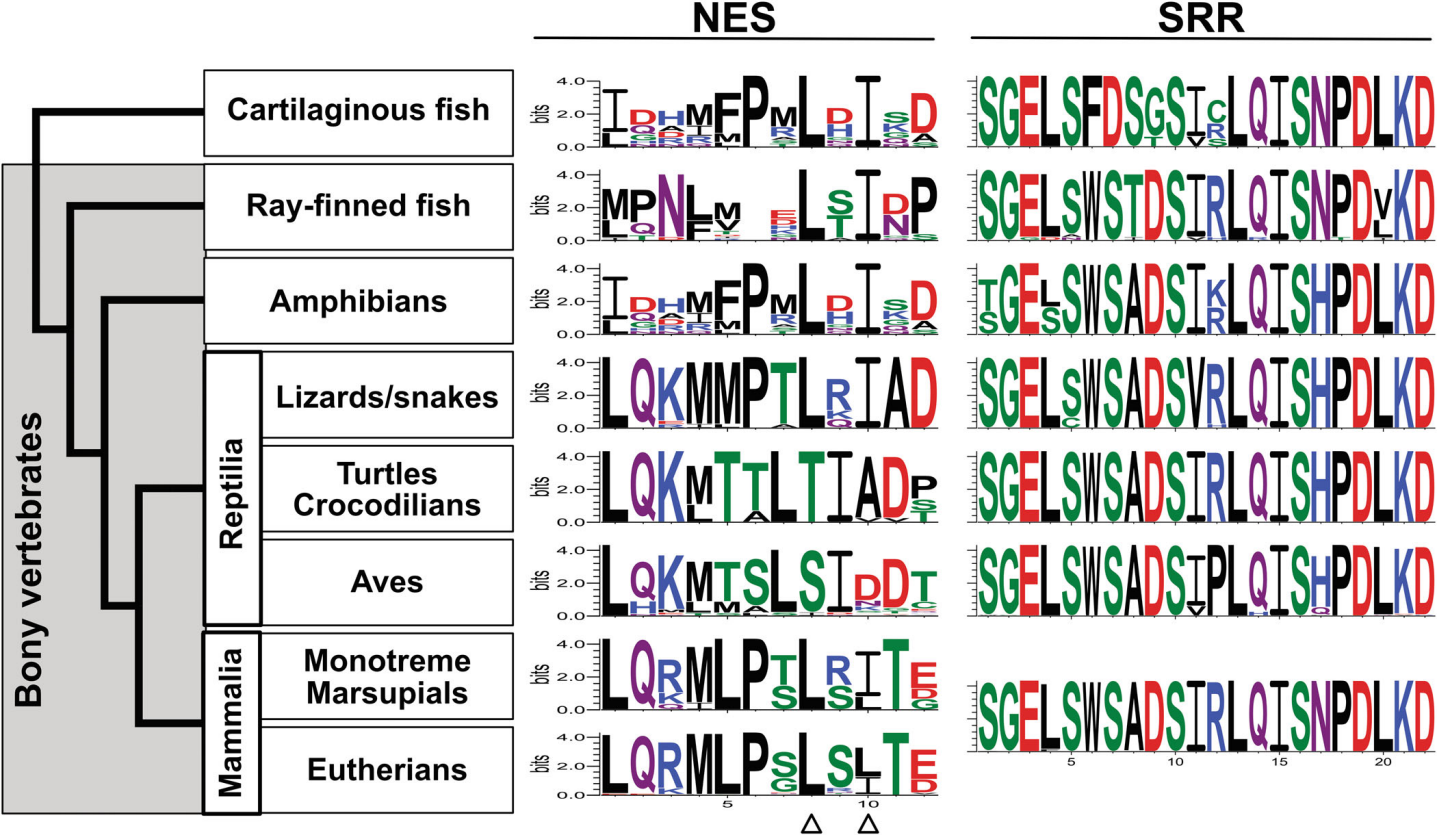
Conserved amino acid sequences found in the IRF5 proteins in jawed animals. The conserved sequences for the NES and the SRR (corresponding to amino acid positions 150−161 and 447−468, respectively, in the human IRF5 NP_001092099.1) are identified from groups of jawed vertebrates and illustrated using sequence logos. The overall height of the stack of letters indicates the sequence conservation at each position. The height of each symbol within each stack indicates the relative frequency of each amino acid. For NES, the two hydrophobic residues shown to be critical to IRF5 nuclear export are indicated by the triangles at the bottom. For SRR, all mammalian ORF5 proteins we examined had identical sequences and shown as a single sequence logo. Each sequence of the conserved regions is shown in Supporting Information S1: Table S2.

To further characterize the functional consequences of the independent evolution identified in IRF proteins, we investigated sequence evolution in these newly diversified IRF subfamilies, focusing on IRF5, a jawed vertebrate‐specific factor whose functional evolution remains poorly understood. The NES and the SRR required for phosphorylation are essential for the functions of IRF5 proteins (Supporting Information S1: Figure S1) [9]. Our comparative analysis of IRF5 sequences across jawed vertebrates revealed strong conservation of both NES and SRR sequences (Figure 2; Supporting Information S1: Table S2). Notably, cartilaginous fish proteins classified as IRF5 in our phylogenetic analysis are annotated as “IRF6‐like” proteins in the database (Figure 1). However, both NES and SRR sequences are conserved in the IRF5 candidate proteins from cartilaginous fishes (Supporting Information S1: Table S2). Structural analysis comparing the IRF5 proteins from human and cartilaginous fish further indicated that these proteins maintain similar structural conformations (Supporting Information S1: Figure S2). These findings demonstrate that key functional sequences governing IRF5 activity were already established before the divergence between cartilaginous fishes (Chondrichthyes) and bony vertebrates (Osteichthyes) and have remained highly conserved across jawed vertebrates, supporting their fundamental importance in IRF5‐mediated immune responses throughout jawed vertebrate evolution.

### 2.3 Emergence of Signal Transducer and Activator of Transcription 2 (STAT2)‐Dependent IRF9 Nuclear‐Cytoplasmic Trafficking in Jawed Vertebrates

IRF9, another IRF protein in the IFN signaling pathway, requires trafficking between nucleus and cytoplasm [17, 18, 19]. Unlike other IRFs, IRF9 lacks an NES but contains a nuclear localization signal (NLS) within its DBD [20]. In IFN signaling, STAT2, which contains a functional NES, interacts with STAT1 and IRF9 to form an IFN‐stimulated gene factor 3 (ISGF3) complex. This complex translocates to the nucleus, where it binds specific DNA sequences and directs the production of interferon‐stimulated genes (ISGs) [20]. In the absence of stimulation, IRF9 constitutively binds STAT2 in the cytoplasm, and this interaction is necessary for its predominant presence in cytoplasm [17, 18, 19]. Without STAT2, IRF9 remains in the nucleus [20, 21].

**Figure 3:**
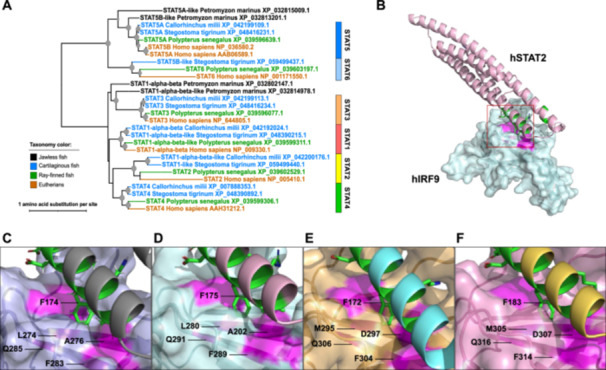
Identification of STAT2 protein candidates from cartilaginous fishes. (A) The maximum likelihood phylogeny among STAT protein families. Internal nodes supported by 80% or higher by both ultrafast bootstraps and SH‐aLRT branch tests are denoted by gray dots with the size correlated with the ultrafast bootstrap values (80%−100%). Terminal branches and sequence names are colored based on the taxonomic groups shown in the color legend. The information on the sequences used is listed in Supporting Information S1: Table S3. (B) Structural modeling of the interacting domains of human IRF9 (hIRF9) and human STAT2 (hSTAT2). IAD of hIRF9 and the coiled‐coil domain (CCD) of hSTAT2 are depicted using a surface model colored in light cyan and a ribbon model colored in light pink, respectively. The area marked with the red box highlights the interaction between a phenylalanine residue from the STAT2 helical bundle and the cleft formed in IRF9. Solved and modeled structures of the STAT2‐IRF9 complex for mouse and two cartilaginous fishes are shown in Supporting Information S1: Figure S3. (C–F) Expanded views of the interaction interface between STAT2 CCD and IRF9 IAD for mouse (C), human (D), *Hypanus sabinus* (E), and *Stegostoma tigrinum* (F)**.** The interactions are observed in the crystal structure of the mouse STAT2‐IRF9 complex (PDB ID: 5OEN) [19]. For humans and the two cartilaginous fishes, the interactions are based on the modeled structures of the STAT2‐IRF9 complex. The key residues involved in the interface are labeled. The phenylalanine (F) on the STAT2 protein is colored in green. The four residues forming the cleft on the IRF9 protein are colored in magenta. The corresponding sequences of the interface area and other details are found in Supporting Information S1: Figure S3.

To understand the evolution of IRF9 cytoplasmic localization in vertebrates, we examined STAT‐family protein distribution across vertebrate lineages. As shown in the phylogeny (Figure 3A), jawless vertebrates (represented by the sea lamprey *Petromyzon marinus*) possess only a limited number of STAT proteins, lacking orthologs for STAT1, 2, 4, and 6. In contrast, cartilaginous fishes (Chondrichthyes) are shown to have orthologs for all STAT proteins. STAT2 candidates identified from cartilaginous fishes are annotated as “STAT1” or “STAT1‐like” proteins in the database. Even though STAT2 proteins show relatively high sequence divergence compared to other STAT family members (indicated by long terminal branch lengths), these cartilaginous fish proteins cluster with representative STAT2 proteins from humans and the Senegal bichir *Polypterus senegalus* with high bootstrap support (100%).

To verify whether these cartilaginous fish STAT2 candidates can bind IRF9 as the canonical vertebrate STAT2 proteins do, we performed structural modeling of the IRF9‐STAT2 binding domains (IRF9‐IAD and STAT2‐CCD) and examined their binding interface (Figure 3B), where the IRF9 protein selectively interacts with STAT2 but not with other STAT proteins [19]. In the solved structure of the mouse protein complex, critical contacts are found between STAT2 residue F174 and four IRF9 residues forming a groove (L274, A276, F283, and Q285) (Figure 3C). These five residues are conserved in both human proteins and the examined cartilaginous fish proteins (Supporting Information S1: Figure S3A). Structural modeling confirms maintenance of this binding interface in the human protein (Figure 3D) as well as in the cartilaginous fish proteins (Figure 3E,F), strongly suggesting these proteins function as STAT2 and maintain cytoplasmic retention of IRF9 through direct binding.

### 2.4 Lineage‐Specific Evolution of IRF5 Signature Sequences in Birds

**Figure 4:**
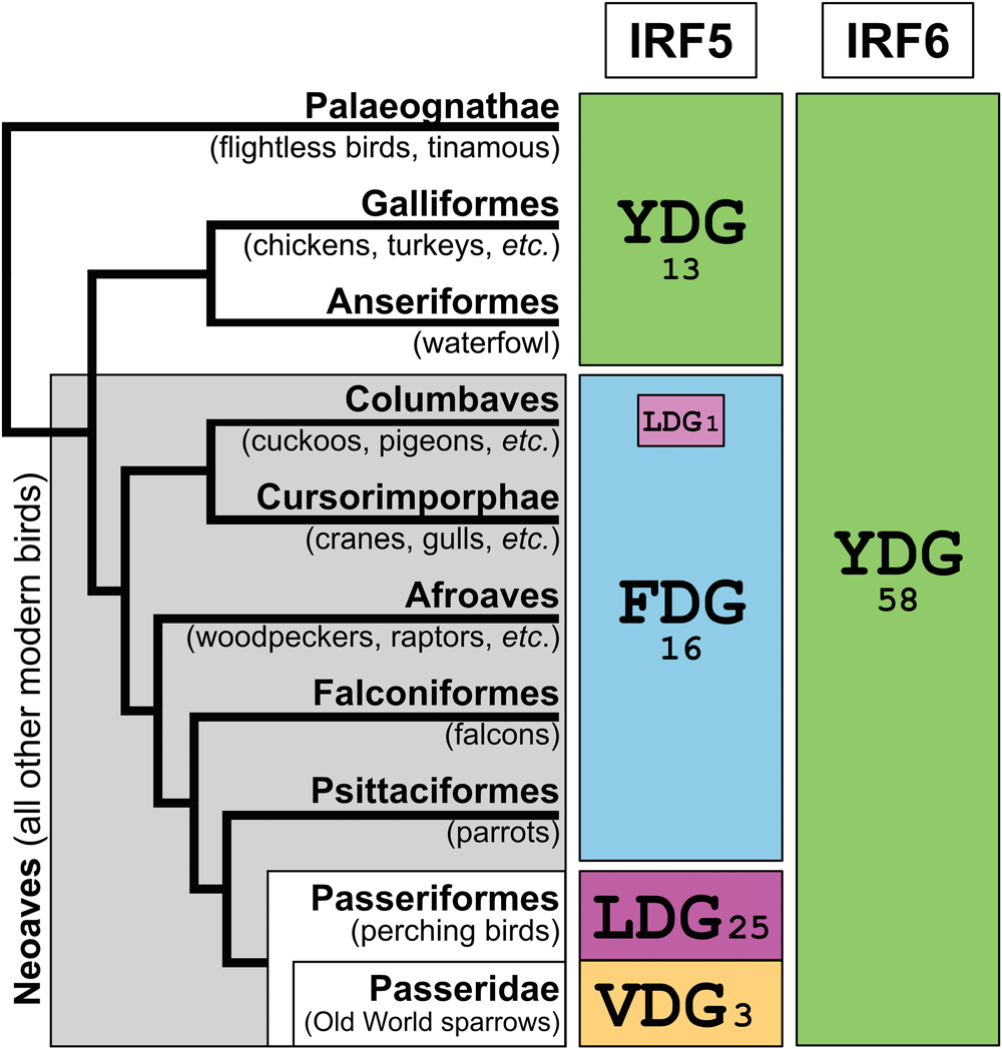
Distribution of YDG motifs in IRF5 and IRF6 among birds. The number shown below or beside each motif is the number of bird species showing the motif. The taxonomic grouping of birds is based on [22]. See Supporting Information S1: Table S4 for all species and sequences used.

The NES sequences of IRF5 in birds and turtles/crocodilians appear like each other but differing from those in other jawed vertebrates (Figure 2 and Supporting Information S1: Table S2). While the overall inflammation and innate immunity systems are similar, birds and mammals differ significantly in the precise composition, diversity, and regulation of their innate immune and inflammatory mechanisms. Previously, we identified YDG as a signature sequence for the IRF5/6 subfamily by comparing DBD regions among nine IRF families [11]. Here, to examine any unique features of IRF5 in birds, we compared IRF5/6 subfamily protein sequences from 58 bird species with those in mammals. As shown in Figure 4 and Supporting Information S1: Table S4, the YDG signature sequence is completely conserved in bird IRF6 proteins. For IRF5, bird species in the lineages diverged early, the Infraclass Palaeognathae and the Superorder Galloanserae [22], maintain the YDG signature sequence. However, in more recently diverged modern bird species (Neoaves), the conserved motif has changed to FDG (or LDG in the common cuckoo *Cuculus canorus*). This FDG signature further changed to LDG in the order Passeriformes (wrens, songbirds, etc.), subsequently changing again to VDG in the species belonging to the family Passeridae (Old World sparrows).

The DBD region of IRF proteins is relatively more conserved. The nearly complete conservation of the YDG motif throughout vertebrate evolution demonstrates strong selective constraints on this motif, suggesting its important functional role in IRF5 proteins across vertebrate lineages. While the substitution of Y to F in the signature motif found in modern bird lineages may have a limited functional impact due to the structural similarity between these residues, the F to L (and V) substitutions may affect DNA‐binding specificity or activity in bird IRF5 proteins. Notably, avian species have evolved unique features in their IRF family composition and regulation [23, 24]. Therefore, further studies are warranted to examine whether these variant bird motifs represent an avian‐specific adaptation that impacts transcriptional networks controlling inflammation and antiviral immunity.

## 3 Discussion

### 3.1 IRF Evolution and the Rise of Nuclear‐Cytoplasmic Trafficking in Vertebrate Immunity

The evolution of IRFs represents a transition from purely nuclear transcription factors to sophisticated signaling molecules with regulated nuclear‐cytoplasmic trafficking. Cartilaginous fishes and the comparison against bony vertebrates provide valuable insights into early vertebrate immune system evolution, including the IFN system components [25].

**Figure 5:**
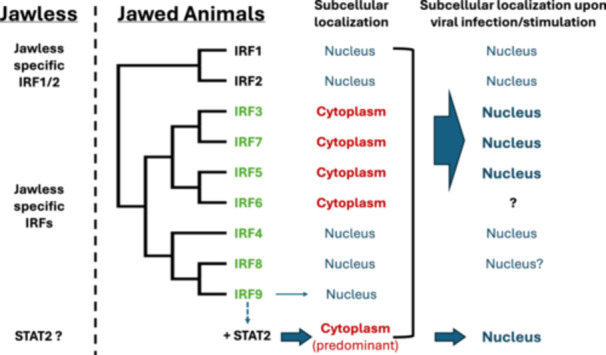
Evolution of the IRF protein family in jawless and jawed vertebrates. Column 1 (from left to right): IRF members in jawless vertebrates. Column 2: IRF members in jawed vertebrates. The IRF members newly expanded in jawed animals are listed in green fonts. Column 3: Subcellular localization of the IRF members in the unstimulated state. The arrow pointing from “+STAT2” indicates STAT2‐dependent cytoplasmic localization of IRF9. Column 4: Subcellular localization of the IRF members in the stimulated state, such as during viral infections and/or IFN treatment.

Our comparative analysis of IRFs between jawless and jawed vertebrates revealed distinct evolutionary patterns in their subcellular localization (Figure 5). While IRF1 and 2 are well‐known nuclear proteins [26, 27], IRF3, 5, 6, and 7 proteins are predominantly cytoplasmic under basal conditions, remaining inactive until stimulation (Figure 5, middle). Upon activation by viral infections or danger signals, IRF3, 5, and 7 proteins undergo nuclear translocation, enabling DNA interaction and transcriptional regulation of immune response genes [5, 6, 28, 29, 30, 31] (Figure 5, right). This cytoplasmic retention serves as a regulatory mechanism, preventing constitutive activation and ensuring controlled immune responses.

Our analysis demonstrates that these cytoplasmic retention mechanisms are functionally conserved for both IRF5 and IRF9 (Figures 2 and 3). IRF3 and IRF7 have been shown to possess NES sequences [20, 31, 32] and their NES sequences were also identified in the cartilaginous fish orthologs (data not shown). Interestingly, although IRF4 and 8 are the closest relatives to IRF9 (Figure 1), they localize mainly to the nucleus [33, 34, 35, 36]. Their apparent lack of physical interaction with STAT2 may explain this nuclear localization.

This nuclear‐to‐cytoplasmic shift accompanied IRF specialization in jawed vertebrates and represents a significant innovation in vertebrate immune regulation, providing several advantages: (1) direct interaction with cytoplasmic danger signals, enabling faster immune responses; (2) new interactions and modifications through cytoplasmic localization; and (3) expanded signaling capabilities through nuclear‐cytoplasmic shuttling. This is particularly relevant for IRF9, as its cytoplasmic retention requires co‐evolution with STAT2 (Figure 3). Through shuttling between the nucleus and cytoplasm, IRFs have evolved from simple transcription factors to sophisticated signal transduction molecules, gaining advantages in response speed, magnitude, detection, and regulation.

The conservation of these nuclear‐cytoplasmic regulatory elements from cartilaginous fishes to mammals including humans reveals fundamental mechanisms underlying viral immunity. These conserved elements serve as key targets for viral manipulation of host immune responses [21, 37, 38, 39]. This evolutionary perspective on IRF regulation offers valuable insights for antiviral therapeutic development and enhances our understanding of host−pathogen interactions in modern virology.

### 3.2 Co‐Evolution of IRF Family Expansion With Jaw Development

The absence of IRF3‐9 homologs in protochordates (e.g., lancelets and tunicates) as well as in jawless fishes supports their specific emergence in jawed vertebrates [11, 13, 14, 16, 40]. This IRF subfamily expansion coincided with the evolution of more sophisticated immune regulation, where precise control of activation is crucial for organism survival. The distinct IRF composition between jawless and jawed vertebrates suggests major evolutionary divergences in immune defense strategies.

Particularly interesting is IRF6, which, despite being predominantly cytoplasmic, functions in development rather than immunity. In humans, IRF6 mutations cause orofacial clefting disorders [41, 42], while mice lacking *irf6* show abnormal jaw, limb, and craniofacial development [43]. These findings suggest IRF6's evolutionary role in jaw development. While both IRF5 and IRF6 appear in cartilaginous fishes, their absence in jawless vertebrates indicates the duplication event occurred after the jawless‐jawed vertebrate divergence. Subsequently, IRF6 must have acquired jaw development functions, while IRF5 maintained immune regulatory roles.

These findings suggest the coordinated evolution of immune regulation and morphological innovation in early vertebrates. The emergence of sophisticated nuclear‐cytoplasmic trafficking coincided with the evolution of jaws and enhanced immune defenses, representing a key advancement in vertebrate immune system evolution. Future studies may reveal additional mechanisms linking spatial protein regulation to the evolution of vertebrate‐specific features.

## 4 Materials and Methods

### 4.1 Searching of IRF and STAT Proteins

The sequences of the human IRF and STAT proteins were used as the queries (see Supporting Information S1: Tables S1 and S3 for the accession numbers). BLASTP protein similarity searches were performed against the nonredundant protein database at the National Center for Biotechnology Information (NCBI) with the default options. When several isoforms were available for a gene, one isoform whose protein sequence was most similar to the human ortholog was selected. Protein sequences that were identical or partial (too short) were excluded. Subfamily classification of IRF and STAT proteins is done based on the phylogenetic placement regardless of the annotation given in the NCBI database. In case of ambiguity, a reciprocal BLATP similarity search was performed further.

For the phylogenetic analyses including all IRF and all STAT subfamilies, protein sequences for each subfamily were chosen from five (four for STATs) species of jawed vertebrates: human *Homo sapiens* (representing eutherians), the Senegal bichir *P. senegalus* (representing ray‐finned fishes), and three (two for STATs) representative cartilaginous fishes including the Australian ghostshark *Callorhinchus milii*, the zebra shark *Stegostoma tigrinum*, as well as the Atlantic stingray *Hypanus sabinus* (only for IRFs). For jawless vertebrates, IRF protein candidates were collected from three representative species: the Far Eastern brook lamprey *Lethenteron reissneri*, the sea lamprey *P. marinus*, and the inshore hagfish *Eptatretus burgeri*. Cartilaginous fish STAT protein candidates were collected only from *P. marinus*. For the IRF4‐like subfamily (also called as IRF10 for fish), no similar sequence was found from humans nor from cartilaginous fishes and consistent sampling was not possible. Thus, for this subfamily, sequences from some other vertebrate species were included. For the NES and conserved signature sequence analyses for IRF5 and IRF6, their protein sequences were collected more broadly across jawed vertebrate lineages. The information of the sequences used (accession numbers and descriptions provided in the database) is listed in Supporting Information S1: Tables S1−S4.

### 4.2 Phylogenetic Analysis and Classification of IRF and STAT Proteins

Multiple sequence alignment of the protein sequences was performed using MAFFT (ver. 7.526) with the E‐INS‐i iterative refinement method [44]. The maximum likelihood (ML) phylogenetic analysis was performed using IQ‐TREE (ver. 1.6.12) with the options for automatic model selection and ultrafast bootstrap analysis and SH‐aLRT branch test for branch support analysis (both with 1000 replicates) [45, 46]. After IRF and STAT subfamily grouping was established, multiple sequence alignment was performed again using only sequences from each subfamily. The final alignment, including all sequences, was performed using the “merge” option with the alignment from each subfamily as the profile using MAFFT. The final ML tree was reconstructed as described above. The visualization of the phylogenies was performed using the Interactive Tree of Life website (ver. 6.9.1) [47].

### 4.3 Identification of Putative NES and SRR

Putative NES sequences were identified across species using the experimentally verified NES sequence in human IRF5 as a reference (NES ID: 87 in NESdb; amino acids 150−161 in UniProt entry Q13568) [48, 49]. Similarly, the SRR sequences across species were identified using the human IRF5 SRR sequence as a reference. Corresponding putative NES and SRR regions in other species' IRF5 sequences were identified through protein sequence alignment with the human IRF5 reference. This approach enabled the evaluation of functional conservation based on both sequence similarity and positional conservation within the IRF5 protein structure. Putative NES sequences were further examined based on the consensus NES pattern: Φ1‐X_2,3_‐Φ2‐X_2,3_‐Φ3‐X‐Φ4, where Φ1~Φ4 represents four hydrophobic residues (L, I, V, F, or M) and X_n_ represents any amino acid with the number (or the range) indicating the spacing requirement between hydrophobic residues [49]. The identified NES and SRR sequences across jawed vertebrate IRF proteins are listed in Supporting Information S1: Table S2.

### 4.4 Sequence Logo

A sequence logo was generated from the protein sequence alignment using WebLogo v3 (https://weblogo.threeplusone.com/create.cgi) [50]. For simplicity, the composition adjustment was suppressed. The amino acid “chemistry” color scheme was chosen.

### 4.5 Protein Three‐Dimensional (3D)‐Structural Analysis

Modeling of protein 3D structures was performed on the AlphaFold3 server (https://alphafoldserver.com) [51]. For IRF5 proteins, modeling was performed using the human (NP_001092099.1) and *C. milii* (XP_007909076.1) sequences. 3D structures were visualized and analyzed with the PyMOL Molecular Graphics System, Version 3.1.0 (Schrödinger LLC, New York, NY, USA). Models with pLDDT scores below 50 indicate low confidence [52] and were excluded from structural comparisons. Similarly, regions with low confidence scores (pLDDT < 50), including domain junctions and carboxyl‐terminal areas, were excluded from the model. The structural similarity was inferred between superimposed models when the root mean square deviation (RMSD) was below 1 Å [53, 54].

To evaluate the interaction potential between STAT2 and IRF9 candidate proteins, structural modeling of these proteins was performed using CCD of STAT2 and IAD of IRF9. For human IRF9 and STAT2 proteins, Q00978 (amino acid positions 219−382 for IAD) and NP_005410.1 (aa 142−315 for CCD), respectively, were used. For cartilaginous fishes, because the IRF9 ortholog candidate was not identified from *C. milii*, the modeling was done using IRF9 and STAT2 candidate proteins from two other species: XP_059807433.1 (aa 224−397 for IAD) and XP_059812301.1 (aa 138−313 for CCD) for *H. sabinus* and XP_048380497.2 (aa 232−408 for IAD) and XP_059499440.1 (aa 149−323 for CCD) for *S. tigrinum*, respectively. The predicted coding sequence of the *S. tigrinum* IRF9 candidate gene (XM_048524540.2) contains an insertion of nucleotide “n” resulting in a codon “X” at aa 258. For the modeling purpose, this position in the *S. tigrinum* protein was replaced with “S” based on the sequences of other cartilaginous fish IRF9 candidate proteins. See Supporting Information S1: Figures S2 and S3 for more detailed description of structural modeling of the IRF5 proteins and the interacting STAT2‐IRF9 domains.

## Author Contributions

Vanessa Hubing, Avery Marquis, and Chanasei Ziemann collected and analyzed the data. Hideaki Moriyama did a structural analysis. Luwen Zhang and Etsuko N. Moriyama conceived the idea, analyzed the data, and wrote the article.

## Conflicts of Interest

The authors declare no conflicts of interest.

## Supporting information

Supporting information.

## Data Availability

The data that supports the findings of this study are available in the Supporting material of this article.

## References

[1] J. E. Darnell Jr. , M. Kerr , and G. R. Stark , “Jak‐STAT Pathways and Transcriptional Activation in Response to IFNs and Other Extracellular Signaling Proteins,” Science 264 (1994): 1415–1421. 8197455 10.1126/science.8197455

[2] K. Honda , A. Takaoka , and T. Taniguchi , “Type I Inteferon Gene Induction by the Interferon Regulatory Factor Family of Transcription Factors,” Immunity 25 (2006): 349–360. 16979567 10.1016/j.immuni.2006.08.009

[3] K. Honda , H. Yanai , H. Negishi , et al., “IRF‐7 Is the Master Regulator of Type‐I Interferon‐Dependent Immune Responses,” Nature 434 (2005): 772–777. 15800576 10.1038/nature03464

[4] T. Tamura , H. Yanai , D. Savitsky , and T. Taniguchi , “The IRF Family Transcription Factors in Immunity and Oncogenesis,” Annual Review of Immunology 26 (2008): 535–584. 10.1146/annurev.immunol.26.021607.090400 18303999

[5] L. Zhang and J. S. Pagano , “Interferon Regulatory Factor 7: A Key Cellular Mediator of LMP‐1 in EBV Latency and Transformation,” Seminars in Cancer Biology 11 (2001): 445–453. 11669606 10.1006/scbi.2001.0411

[6] L. Zhang and J. S. Pagano , “Review: Structure and Function of IRF‐7,” Journal of Interferon & Cytokine Research 22 (2002): 95–101. 11846980 10.1089/107999002753452700

[7] B. J. Barnes , J. Richards , M. Mancl , S. Hanash , L. Beretta , and P. M. Pitha , “Global and Distinct Targets of IRF‐5 and IRF‐7 During Innate Response to Viral Infection,” Journal of Biological Chemistry 279 (2004): 45194–45207. 15308637 10.1074/jbc.M400726200

[8] A. Takaoka , H. Yanai , S. Kondo , et al., “Integral Role of IRF‐5 in the Gene Induction Programme Activated by Toll‐Like Receptors,” Nature 434 (2005): 243–249. 15665823 10.1038/nature03308

[9] H. L. Eames , A. L. Corbin , and I. A. Udalova , “Interferon Regulatory Factor 5 in Human Autoimmunity and Murine Models of Autoimmune Disease,” Translational Research 167 (2016): 167–182. 26207886 10.1016/j.trsl.2015.06.018

[10] D. A. Savitsky , H. Yanai , T. Tamura , T. Taniguchi , and K. Honda , “Contribution of IRF5 in B Cells to the Development of Murine SLE‐Like Disease Through Its Transcriptional Control of the IgG2a Locus,” Proceedings of the National Academy of Sciences of the United States of America 107 (2010): 10154–10159. 20479222 10.1073/pnas.1005599107 PMC2890425

[11] M. Angeletti , W. L. N. Hsu , N. Majo , H. Moriyama , E. N. Moriyama , and L. Zhang , “Adaptations of Interferon Regulatory Factor 3 With Transition From Terrestrial to Aquatic Life,” Scientific Reports 10 (2020): 4508. 32161340 10.1038/s41598-020-61365-9 PMC7066157

[12] K. Du , Z. Zhong , C. Fang , et al., “Ancient Duplications and Functional Divergence in the Interferon Regulatory Factors of Vertebrates Provide Insights Into the Evolution of Vertebrate Immune Systems,” Developmental & Comparative Immunology 81 (2018): 324–333. 29253557 10.1016/j.dci.2017.12.016

[13] B. Huang , Z. T. Qi , Z. Xu , and P. Nie , “Global Characterization of Interferon Regulatory Factor (IRF) Genes in Vertebrates: Glimpse of the Diversification in Evolution,” BMC Immunology 11 (2010): 22. 20444275 10.1186/1471-2172-11-22 PMC2885996

[14] J. Nehyba , R. Hrdlickova , and H. R. Bose , “Dynamic Evolution of Immune System Regulators: The History of the Interferon Regulatory Factor Family,” Molecular Biology and Evolution 26 (2009): 2539–2550. 19638535 10.1093/molbev/msp167 PMC2767096

[15] D. Qi , Y. Chao , J. Liang , et al., “Adaptive Evolution of Interferon Regulatory Factors Is Not Correlated With Body Scale Reduction or Loss in Schizothoracine Fish,” Fish & Shellfish Immunology 73 (2018): 145–151. 29246809 10.1016/j.fsi.2017.12.013

[16] S. Drury , G. Claussen , A. Zetterman , H. Moriyama , E. N. Moriyama , and L. Zhang , “Evolution and Emergence of Primate‐Specific Interferon Regulatory Factor 9,” Journal of Medical Virology 95 (2023): e28521. 36691924 10.1002/jmv.28521 PMC10107944

[17] G. Banninger and N. C. Reich , “STAT2 Nuclear Trafficking,” Journal of Biological Chemistry 279 (2004): 39199–39206. 15175343 10.1074/jbc.M400815200

[18] K. Fink and N. Grandvaux , “STAT2 and IRF9: Beyond ISGF3,” JAK‐STAT 2 (2013): e27521. 24498542 10.4161/jkst.27521 PMC3906322

[19] S. Rengachari , S. Groiss , J. M. Devos , E. Caron , N. Grandvaux , and D. Panne , “Structural Basis of STAT2 Recognition by IRF9 Reveals Molecular Insights Into ISGF3 Function,” Proceedings of the National Academy of Sciences of the United States of America 115 (2018): E601–E609. 29317535 10.1073/pnas.1718426115 PMC5789952

[20] J. F. Lau , J. P. Parisien , and C. M. Horvath , “Interferon Regulatory Factor Subcellular Localization Is Determined by a Bipartite Nuclear Localization Signal in the DNA‐Binding Domain and Interaction With Cytoplasmic Retention Factors,” Proceedings of the National Academy of Sciences of the United States of America 97 (2000): 7278–7283. 10860992 10.1073/pnas.97.13.7278 PMC16536

[21] A. Paul , T. H. Tang , and S. K. Ng , “Interferon Regulatory Factor 9 Structure and Regulation,” Frontiers in Immunology 9 (August 2018): 01831, 10.3389/fimmu.2018.01831. PMC6095977 30147694

[22] J. Stiller , S. Feng , A.‐A. Chowdhury , et al., “Complexity of Avian Evolution Revealed by Family‐Level Genomes,” Nature 629 (2024): 851–860. 38560995 10.1038/s41586-024-07323-1 PMC11111414

[23] J. Mountford , A. Gheyas , L. Vervelde , and J. Smith , “Genetic Variation in Chicken Interferon Signalling Pathway Genes in Research Lines Showing Differential Viral Resistance,” Animal Genetics 53 (2022): 640–656. 35739459 10.1111/age.13233 PMC9544680

[24] L. Ungrová , J. Geryk , M. Kohn , et al., “Avian Interferon Regulatory Factor (IRF) Family Reunion: IRF3 and IRF9 Found,” *bioRxiv* (2024). 10.1186/s12915-025-02261-4 PMC12220609 40597257

[25] C. J. Secombes and J. Zou , “Evolution of Interferons and Interferon Receptors,” Frontiers in Immunology 8 (2017): 209. 28303139 10.3389/fimmu.2017.00209 PMC5332411

[26] F. Schaper , S. Kirchhoff , G. Posern , et al., “Functional Domains of Interferon Regulatory Factor I (IRF‐1),” Biochemical Journal 335, no. pt. 1 (1998): 147–157. 9742224 10.1042/bj3350147 PMC1219763

[27] A. A. Vora , P. K. Mondala , C. Costello , A. R. MacLeod , and L. A. Crews , “Sensitive Intranuclear Flow Cytometric Quantification of IRF4 Protein in Multiple Myeloma and Normal Human Hematopoietic Cells,” STAR Protocols 2 (2021): 100565. 34136833 10.1016/j.xpro.2021.100565 PMC8176358

[28] A. Glanz , S. Chakravarty , M. Varghese , et al., “Transcriptional and Non‐Transcriptional Activation, Posttranslational Modifications, and Antiviral Functions of Interferon Regulatory Factor 3 and Viral Antagonism by the SARS‐Coronavirus,” Viruses 13 (2021): 575. 33805458 10.3390/v13040575 PMC8066409

[29] J. Hiscott , P. Pitha , P. Genin , et al., “Triggering the Interferon Response: The Role of IRF‐3 Transcription Factor,” Journal of Interferon & Cytokine Research 19 (1999): 1–13. 10048763 10.1089/107999099314360

[30] C. A. Jefferies , “Regulating IRFs in IFN Driven Disease,” Frontiers in Immunology 10 (2019): 325. 30984161 10.3389/fimmu.2019.00325 PMC6449421

[31] S. Ning , J. S. Pagano , and G. N. Barber , “IRF7: Activation, Regulation, Modification and Function,” Genes & Immunity 12 (2011): 399–414. 21490621 10.1038/gene.2011.21 PMC4437765

[32] R. Lin , Y. Mamane , and J. Hiscott , “Multiple Regulatory Domains Control IRF‐7 Activity in Response to Virus Infection,” Journal of Biological Chemistry 275 (2000): 34320–34327. 10893229 10.1074/jbc.M002814200

[33] L. Laricchia‐Robbio , T. Tamura , T. Karpova , B. L. Sprague , J. G. McNally , and K. Ozato , “Partner‐Regulated Interaction of IFN Regulatory Factor 8 With Chromatin Visualized in Live Macrophages,” Proceedings of the National Academy of Sciences of the United States of America 102 (2005): 14368–14373. 16183743 10.1073/pnas.0504014102 PMC1242294

[34] S. Marecki and M. J. Fenton , “Review: The Role of IRF‐4 in Transcriptional Regulation,” Journal of Interferon & Cytokine Research 22 (2002): 121–133. 11846983 10.1089/107999002753452737

[35] H. Minderman , O. Maguire , K. L. O'Loughlin , J. Muhitch , P. K. Wallace , and S. I. Abrams , “Total Cellular Protein Presence of the Transcription Factor IRF8 Does Not Necessarily Correlate With Its Nuclear Presence,” Methods 112 (2017): 84–90. 27582125 10.1016/j.ymeth.2016.08.011 PMC5545900

[36] J. Schönheit , C. Kuhl , M. L. Gebhardt , et al., “PU.1 Level‐Directed Chromatin Structure Remodeling at the Irf8 Gene Drives Dendritic Cell Commitment,” Cell Reports 3 (2013): 1617–1628. 23623495 10.1016/j.celrep.2013.04.007

[37] H. S. Chiang and H. M. Liu , “The Molecular Basis of Viral Inhibition of IRF‐ and STAT‐Dependent Immune Responses,” Frontiers in Immunology 9 (2019): 3086. 30671058 10.3389/fimmu.2018.03086 PMC6332930

[38] N. C. Reich , “Review: Nuclear/Cytoplasmic Localization of IRFs in Response to Viral Infection or Interferon Stimulation,” Journal of Interferon & Cytokine Research 22 (2002): 103–109. 11846981 10.1089/107999002753452719

[39] H. Schwanke , M. Stempel , and M. M. Brinkmann , “Of Keeping and Tipping the Balance: Host Regulation and Viral Modulation of IRF3‐Dependent IFNB1 Expression,” Viruses 12 (2020): 733. 32645843 10.3390/v12070733 PMC7411613

[40] J. Kasamatsu , H. Oshiumi , M. Matsumoto , M. Kasahara , and T. Seya , “Phylogenetic and Expression Analysis of Lamprey Toll‐Like Receptors,” Developmental & Comparative Immunology 34 (2010): 855–865. 20363250 10.1016/j.dci.2010.03.004

[41] S. Kondo , B. C. Schutte , R. J. Richardson , et al., “Mutations in IRF6 Cause Van Der Woude and Popliteal Pterygium Syndromes,” Nature Genetics 32 (2002): 285–289. 12219090 10.1038/ng985 PMC3169431

[42] T. M. Zucchero , M. E. Cooper , B. S. Maher , et al., “Interferon Regulatory Factor 6 (IRF6) Gene Variants and the Risk of Isolated Cleft Lip or Palate,” New England Journal of Medicine 351 (2004): 769–780. 15317890 10.1056/NEJMoa032909

[43] C. R. Ingraham , A. Kinoshita , S. Kondo , et al., “Abnormal Skin, Limb and Craniofacial Morphogenesis in Mice Deficient for Interferon Regulatory Factor 6 (Irf6),” Nature Genetics 38 (2006): 1335–1340. 17041601 10.1083/ng1903 PMC2082114

[44] K. Katoh and D. M. Standley , “MAFFT Multiple Sequence Alignment Software Version 7: Improvements in Performance and Usability,” Molecular Biology and Evolution 30 (2013): 772–780. 23329690 10.1093/molbev/mst010 PMC3603318

[45] S. Kalyaanamoorthy , B. Q. Minh , T. K. F. Wong , A. von Haeseler , and L. S. Jermiin , “ModelFinder: Fast Model Selection for Accurate Phylogenetic Estimates,” Nature Methods 14 (2017): 587–589. 28481363 10.1038/nmeth.4285 PMC5453245

[46] L.‐T. Nguyen , H. A. Schmidt , A. von Haeseler , and B. Q. Minh , “IQ‐TREE: A Fast and Effective Stochastic Algorithm for Estimating Maximum‐Likelihood Phylogenies,” Molecular Biology and Evolution 32 (2014): 268–274. 25371430 10.1093/molbev/msu300 PMC4271533

[47] I. Letunic and P. Bork , “Interactive Tree of Life (iTOL) v6: Recent Updates to the Phylogenetic Tree Display and Annotation Tool,” Nucleic Acids Research 52 (2024): W78–W82. 38613393 10.1093/nar/gkae268 PMC11223838

[48] T. la Cour , L. Kiemer , A. Mølgaard , R. Gupta , K. Skriver , and S. Brunak , “Analysis and Prediction of Leucine‐Rich Nuclear Export Signals,” Protein Engineering, Design and Selection 17 (2004): 527–536. 10.1093/protein/gzh062 15314210

[49] D. Xu , A. Farmer , G. Collett , N. V. Grishin , and Y. M. Chook , “Sequence and Structural Analyses of Nuclear Export Signals in the NESdb Database,” Molecular Biology of the Cell 23 (2012): 3677–3693. 22833565 10.1091/mbc.E12-01-0046 PMC3442415

[50] G. E. Crooks , G. Hon , J. M. Chandonia , and S. E. Brenner , “WebLogo: A Sequence Logo Generator,” Genome Research 14 (2004): 1188–1190. 15173120 10.1101/gr.849004 PMC419797

[51] J. Abramson , J. Adler , J. Dunger , et al., “Accurate Structure Prediction of Biomolecular Interactions With AlphaFold 3,” Nature 630 (2024): 493–500. 38718835 10.1038/s41586-024-07487-w PMC11168924

[52] V. Mariani , M. Biasini , A. Barbato , and T. Schwede , “lDDT: A Local Superposition‐Free Score for Comparing Protein Structures and Models Using Distance Difference Tests,” Bioinformatics 29 (2013): 2722–2728. 23986568 10.1093/bioinformatics/btt473 PMC3799472

[53] S. Ghosh , V. Gadiyaram , and S. Vishveshwara , “Validation of Protein Structure Models Using Network Similarity Score,” Proteins: Structure, Function, and Bioinformatics 85 (2017): 1759–1776. 10.1002/prot.25332 28598579

[54] I. Kufareva and R. Abagyan , “Methods of Protein Structure Comparison,” Methods in Molecular Biology 857 (2012): 231–257. 22323224 10.1007/978-1-61779-588-6_10 PMC4321859

