# Supplementary material for: Cytoplasmic Shift of Interferon Regulatory Factors Co‐Evolved With Jawed Vertebrate Innate Immunity
Source: J Med Virol. 2025 Feb 20;97(2):e70247. doi: 10.1002/jmv.70247 (PMC11841930; doi:10.1002/jmv.70247)
Supplement: Supplementary file 1 — Supporting information. [file JMV-97-e70247-s001.pdf]

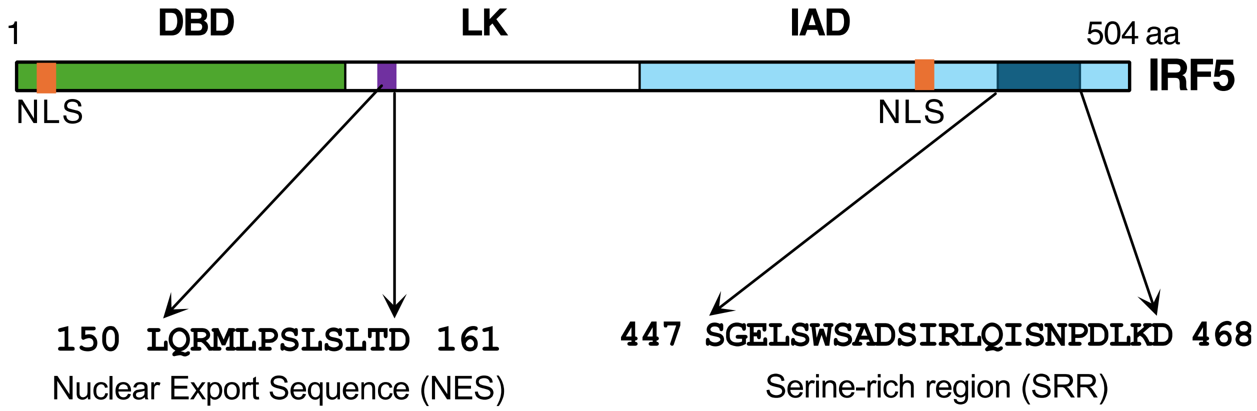

Supplemental Figure S1. Domain structure of IRF5 protein. The DNA-binding domain (DBD), the IRF-association domain (IAD), the linker region (LK), serine-rich region (SRR), nuclear localization signal (NLS) and nuclear export signal (NES) are as shown. The amino acid sequences shown for NES and SRR are from the human IRF5 (NP_001092099.1).

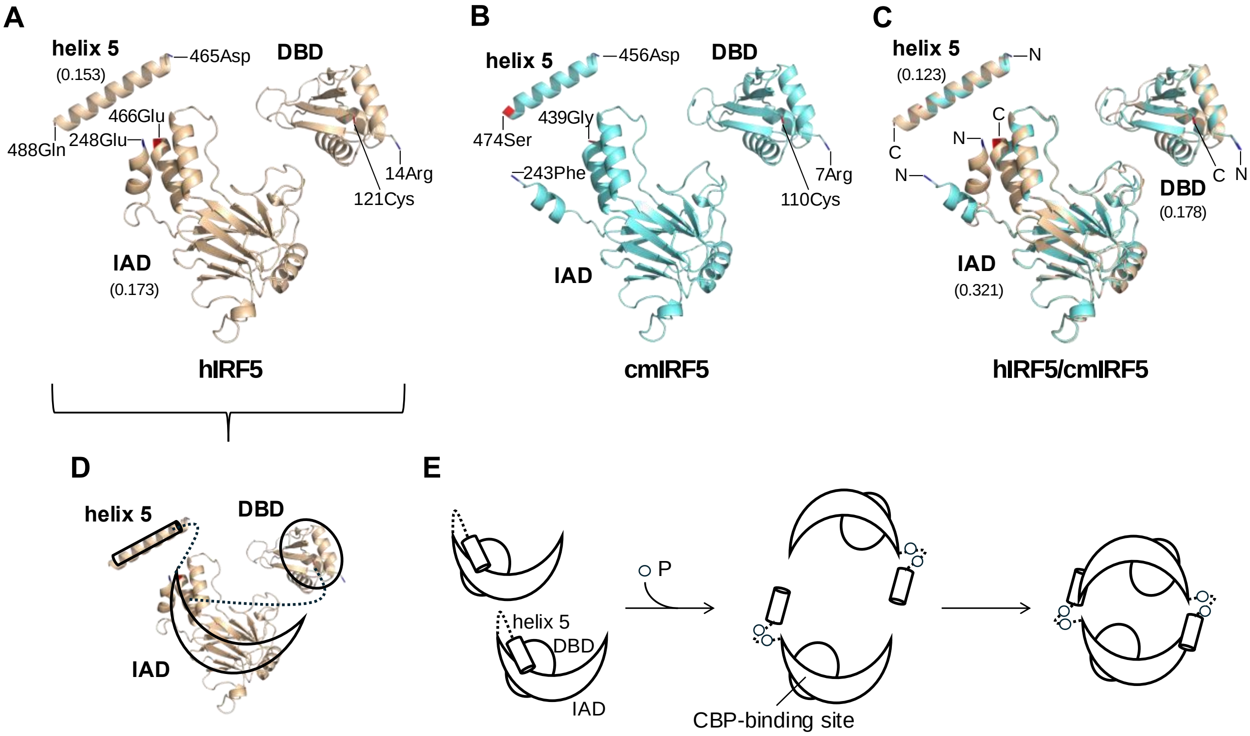

Supplemental Figure S2. Structural modeling of the *Callorhinchus milii* IRF5 protein candidate. A. Cartoon models of human IRF5 (hIRF5) domains generated by AlphaFold3. For the core IAD and helix 5, RMSD values relative to the hIRF5 crystal structure (PDB ID: 3DSH) are indicated in parentheses. The DBD does not have a corresponding experimental structure. **B. Cartoon models of *C. milii* IRF5 protein candidate (cmIRF5) domains generated by AlphaFold3. C. Superimposed domain models of hIRF5 and cmIRF5 generated by AlphaFold3.** For each domain, the RMSD value (in angstroms, Å) between the two models is indicated in parentheses. RMSD values below 1 Å indicate strong structural similarity. The N-terminal helical structure in the IAD was excluded from superimposition. **D. Schematic representation of the hIRF5 domains.** Following (1), from the N-terminus, DBD, the IAD domain, and the dimerization helix 5 are depicted as an oval, a crescent, and a cylinder, respectively, with connecting loops shown as dotted lines. **E. Phosphorylation induced structural transition and dimerization of IRF5.** When unphosphorylated, DBD is located behind the core IAD, with helix 5 retracted into the IAD (1). Unphosphorylated IRF5 remains in the cytoplasm. Phosphorylated IRF5 extends helix 5, enabling dimerization with another IRF protein and also exposing the CREB-binding protein (CBP)-binding site (Phosphates, P, are represented as circles in the diagram). Phosphorylated IRF5 dimers translocate into the nucleus. With the structural similarity maintained in all three domains, we hypothesize that cmIRF5 can adopt a domain conformation enabling a dimer-containing function, similar to that observed in hIRF5 (1). Thus, cmIRF5 is considered to perform the same function as hIRF5.

1. Chen W, Lam SS, Srinath H, Jiang Z, Correia JJ, Schiffer CA, Fitzgerald KA, Lin K, Royer WE Jr. Insights into interferon regulatory factor activation from the crystal structure of dimeric IRF5. Nat Struct Mol Biol. 2008 Nov;15(11):1213-20.

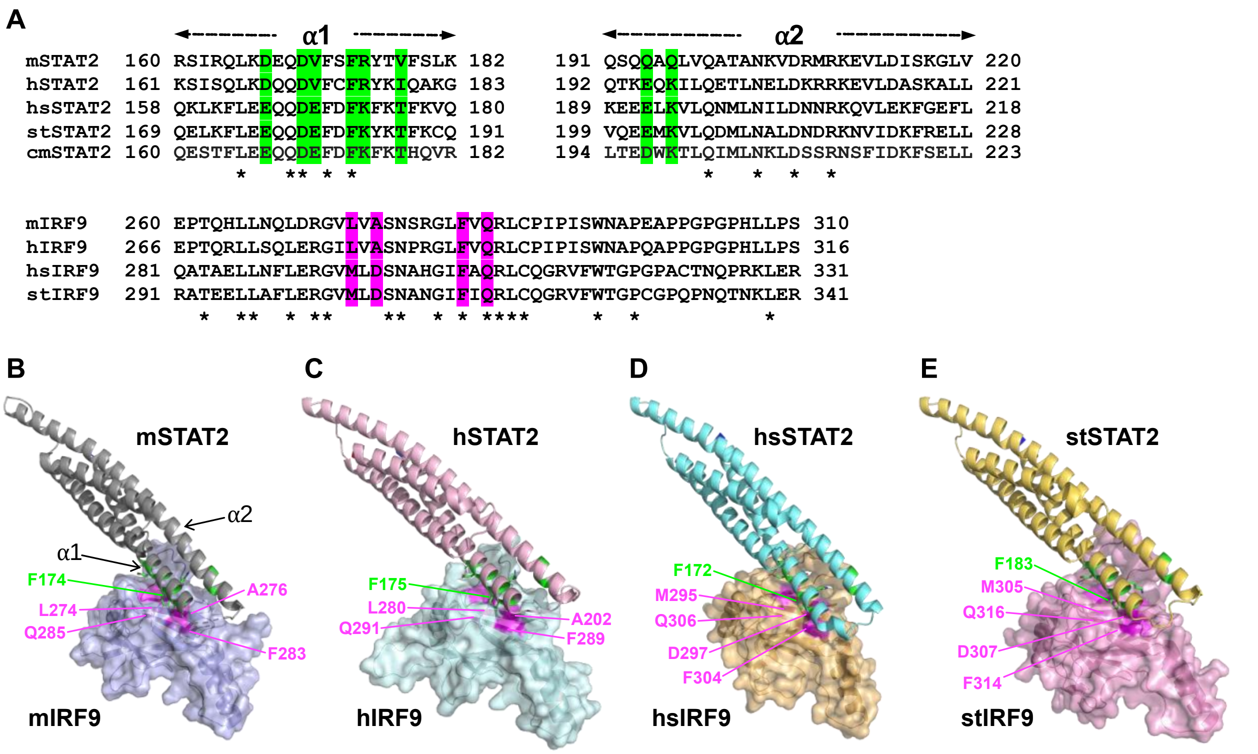

Supplemental Figure S3. Structural modeling of the STAT2-IRF9 complex and evaluation of the interaction interface. A. Amino acid residues involved in the complex formation of STAT2 CCD and IRF9 IAD. Sequences are for mouse (mSTAT2 and mIRF9), human (hSTAT2 and hIRF9), *Hypanus sabinus* (hsSTAT2 and hsIRF9), *Stegostoma tigrinum* (stSTAT2 and stIRF9), and *Callorhinchus milii* (cmSTAT2). Key residues are highlighted in green (for STAT2) and magenta (for IRF9). 'α1' and 'α2' above the STAT2 alignment indicates these two regions are part of the two a -helices. Fully conserved positions are indicated by '*' at the bottom. **B. The crystal structure of the mouse STAT2-IRF9 complex (PDB ID: 5OEN). C-E. Structural modeling of the IRF9-STAT2 complex using the human and two cartilaginous fish proteins.** IAD of IRF9 and CCD of STAT2 are depicted using a surface model and a ribbon model, respectively. The key residues at the interface are highlighted where the four conserved IRF9 residues (shown in magenta) form a groove, which accommodates the STAT2 phenylalanine (*e.g.*, F174 in B shown in green).

Detailed method: The methods and sequences used for structural modeling are described in the Materials and Methods of the main text. The modeled structures of the IRF9 IAD and the STAT2 CCD from human and the two cartilaginous fish species showed strong similarity to the corresponding domains in the crystal structure of the mouse complex (PDB ID: 5OEN), with RMSD values < 1 Å. When the entire STAT2 and IRF9 protein sequences were used for modeling with AlphaFold 3, the resulted model yielded predicted template modeling (pTM) scores above 0.5 across all species, indicating that the overall fold of the complex is similar to the template structure (1,2). However, the association of the STAT2 and IRF9 subunits in the overall complex model had an interface predicted template modeling (ipTM) score ranging from 0.5 to 0.6, indicating moderate accuracy in the predicted relative positions of the subunits. To address this, the STAT2 CCD and IRF9 IAD were isolated and re-evaluated with ZDOCK (3), which confirmed that a complex resembling the crystal structure was feasible. Therefore, the model demonstrating the binding between STAT2 and IRF9 was considered valid.

1. Zhang Y, Skolnick J. Scoring function for automated assessment of protein structure template quality. Proteins. 2004;57(4):702-10.
2. Xu J, Zhang Y. How significant is a protein structure similarity with TM-score = 0.5? Bioinformatics. 2010;26(7):889-95.
3. Pierce BG, Wiehe K, Hwang H, Kim BH, Vreven T, Weng Z. ZDOCK server: interactive docking prediction of protein-protein complexes and symmetric multimers. Bioinformatics. 2014;30(12):1771-3.

Table S1. IRF potein sequences used for Figure 1.[a]

[a]IRF family classification is based on the phylogenetic placement. Sequences with ambiguous or incorrect annotations are shown with yellow background.

			IRF1		IRF2	
**Species**	**Common name**	**Order (subgroup)**	**Accession #**	**Description**	**Accession #**	**Description**
**[Cartilaginous fish]**						
Callorhinchus milii	Australian ghostshark	Chimaeriformes	XP_042195973.1	interferon regulatory factor 1 isoform X1	XP_007905076.1	interferon regulatory factor 2 isoform X1
			XP_007902735.2	interferon regulatory factor 1		
Stegostoma tigrinum	Zebra shark	Orectolobiformes	XP_048399930.1	interferon regulatory factor 1-like	XP_048383526.1	interferon regulatory factor 2 isoform X2
Hypanus sabinus	Atlantic stingray	Myliobatiformes	XP_059846575.1	interferon regulatory factor 1-like	XP_059830336.1	interferon regulatory factor 2 isoform X1
**[Ray-finned fish]**						
Polypterus senegalus	Senegal bichir	Polypteriformes	XP_039630608.1	interferon regulatory factor 1b isoform X1	XP_039606956.1	interferon regulatory factor 2
**[Eutherians]**						
Homo sapiens	Human	Primate (great apes)	NP_002189.1	interferon regulatory factor 1 isoform 1	NP_002190.2	interferon regulatory factor 2

			IRF3		IRF7	
**Species**	**Common name**	**Order (subgroup)**	**Accession #**	**Description**	**Accession #**	**Description**
**[Cartilaginous fish]**						
Callorhinchus milii	Australian ghostshark	Chimaeriformes	XP_042202476.1	interferon regulatory factor 3	XP_007907546.1	interferon regulatory factor 7
Stegostoma tigrinum	Zebra shark	Orectolobiformes	XP_048377578.1	interferon regulatory factor 3	XP_048401646.1	interferon regulatory factor 7 isoform X1
Hypanus sabinus	Atlantic stingray	Myliobatiformes	XP_059809532.1	interferon regulatory factor 3-like isoform X3	XP_059832250.1	interferon regulatory factor 3-like
**[Ray-finned fish]**						
Polypterus senegalus	Senegal bichir	Polypteriformes	XP_039629766.1	interferon regulatory factor 3-like	XP_039600445.1	interferon regulatory factor 7
**[Eutherians]**						
Homo sapiens	Human	Primate (great apes)	NP_001562.1	interferon regulatory factor 3 isoform 1	AAC70999.1	interferon regulatory factor 7H

			IRF5		IRF6	
**Species**	**Common name**	**Order (subgroup)**	**Accession #**	**Description**	**Accession #**	**Description**
**[Cartilaginous fish]**						
Callorhinchus milii	Australian ghostshark	Chimaeriformes	XP_007909076.1	interferon regulatory factor 6	XP_007897748.1	interferon regulatory factor 6
Stegostoma tigrinum	Zebra shark	Orectolobiformes	XP_048409947.1	interferon regulatory factor 6-like isoform X1	XP_048409197.1	interferon regulatory factor 6
Hypanus sabinus	Atlantic stingray	Myliobatiformes	XP_059842980.1	interferon regulatory factor 6-like	XP_059805988	interferon regulatory factor 6
**[Ray-finned fish]**						
Polypterus senegalus	Senegal bichir	Polypteriformes	XP_039617364.1	interferon regulatory factor 5	XP_039604520.1	interferon regulatory factor 6
**[Eutherians]**						
Homo sapiens	Human	Primate (great apes)	NP_001092099.1	interferon regulatory factor 5 isoform d	NP_006138.1	interferon regulatory factor 6 isoform 1

			IRF4		IRF4-like	
**Species**	**Common name**	**Order (subgroup)**	**Accession #**	**Description**	**Accession #**	**Description**
**[Cartilaginous fish]**						
Callorhinchus milii	Australian ghostshark	Chimaeriformes	XP_007887806.1	interferon regulatory factor 4a isoform X1		
Stegostoma tigrinum	Zebra shark	Orectolobiformes	XP_048415031.1	interferon regulatory factor 4-like		
Hypanus sabinus	Atlantic stingray	Myliobatiformes	XP_059801297.1	interferon regulatory factor 4a		
**[Ray-finned fish]**						
Polypterus senegalus	Senegal bichir	Polypteriformes	XP_039609366.1	interferon regulatory factor 4a	XP_039591728.1	interferon regulatory factor 10
**[Amphibians]**						
Xenopus tropicalis	Western clawed frog	Anura			XP_031750663.1	interferon regulatory factor 4-like isoform X2
**[Squamata]**						
Python bivittatus					XP_007442483.1	interferon regulatory factor 4-like, partial
**[Archosauriformes]**						
Gallus gallus	Red junglefowl	Galliformes			NP_989889.1	interferon regulatory factor 9
**[Eutherians]**						
Homo sapiens	Human	Primate (great apes)	NP_002451.2	interferon regulatory factor 4 isoform 1		
Propithecus coquereli	Coquerel's sifaka	Primates (prosimians)			XP_012493186.1	interferon regulatory factor 4-like
Canis lupus familiaris	Dog	Carnivora (canid)			XP_038289371.1	interferon regulatory factor 4-like

			IRF8		IRF9	
**Species**	**Common name**	**Order (subgroup)**	**Accession #**	**Description**	**Accession #**	**Description**
**[Cartilaginous fish]**						
Callorhinchus milii	Australian ghostshark	Chimaeriformes	XP_007887425.1	interferon regulatory factor 8		
Stegostoma tigrinum	Zebra shark	Orectolobiformes	XP_048402935.1	interferon regulatory factor 8	XP_048380497.2	LOW QUALITY PROTEIN: interferon regulatory factor 8-like
Hypanus sabinus	Atlantic stingray	Myliobatiformes	XP_059849182.1	interferon regulatory factor 8	XP_059807433.1	interferon regulatory factor 8-like isoform X1
**[Ray-finned fish]**						
Polypterus senegalus	Senegal bichir	Polypteriformes	XP_039619327.1	interferon regulatory factor 8 isoform X1	XP_039603616.1	interferon regulatory factor 9
**[Eutherians]**						
Homo sapiens	Human	Primate (great apes)	NP_001350836.1	interferon regulatory factor 8 isoform 1	NP_006075.3	interferon regulatory factor 9 isoform 2

			JL-IRF I		JL-IRF II	
**Species**	**Common name**	**Order (subgroup)**	**Accession #**	**Description**	**Accession #**	**Description**
**[Jawless fish]**						
Lethenteron reissneri	Far Eastern brook Lamprey	Petromyzontiformes	XP_061421088.1	interferon regulatory factor 2	XP_061434089.1	putative uncharacterized protein DDB_G0291608 isoform X1
					XP_061434090.1	interferon regulatory factor 1-like isoform X2
Petromyzon marinus	Sea lamprey	Petromyzontiformes	XP_032805773.1	uncharacterized protein LOC116940282 isoform X1	XP_032834583.1	interferon regulatory factor 2-like

			JL-IRF III		JL-IRF IV	
**Species**	**Common name**	**Order (subgroup)**	**Accession #**	**Description**	**Accession #**	**Description**
**[Jawless fish]**						
Lethenteron reissneri	Far Eastern brook Lamprey	Petromyzontiformes	XP_061434999.1	interferon regulatory factor 6-like		
			XP_061420173.1	interferon regulatory factor 5-like		
			XP_061435733.1	interferon regulatory factor 5-like isoform X1		
			XP_061435561.1	interferon regulatory factor 5-like isoform X2		
Petromyzon marinus	Sea lamprey	Petromyzontiformes	XP_032833984.1	interferon regulatory factor 6-like isoform X1		
			XP_032805220.1	interferon regulatory factor 5-like isoform X2		
			XP_032834195.1	interferon regulatory factor 4-like	XP_032816081.1	interferon regulatory factor 6-like

			JL-IRF V	
**Species**	**Common name**	**Order (subgroup)**	**Accession #**	**Description**
**[Jawless fish]**				
Lethenteron reissneri	Far Eastern brook Lamprey	Petromyzontiformes	XP_061421764.1	interferon regulatory factor 8-like
			XP_061429197.1	interferon regulatory factor 8-like
			XP_061431988.1	interferon regulatory factor 4-like
			XP_061432107.1	interferon regulatory factor 4-like isoform X1
			XP_061437984.1	uncharacterized protein LOC133363000 isoform X1
			XP_061403076.1	interferon regulatory factor 9-like
Petromyzon marinus	Sea lamprey	Petromyzontiformes	XP_032830386.1	interferon regulatory factor 4-like
			XP_032833405.1	interferon regulatory factor 8-like isoform X1
			XP_032833262.1	interferon regulatory factor 8-like
			XP_032834870.1	uncharacterized protein LOC116957060
Eptatretus burgeri	Inshore hagfish	Myxiniformes	WGF17116.1	IRF-like protein, partial

Table S2. IRF5 potein sequences used to identify the sequences of NES and serine-rich region of the activation domain.

Species	Order	Accession #	Description	Potential NES*	Potential SRR
**[Cartilaginous fish]**					
Stegostoma fasciatum	Orectolobiformes	XP_048409947.1	interferon regulatory factor 6-like isoform X1	LQALFPSMTLED	SGELSFDSGSICLQISNPDLKD
Chiloscyllium punctatum	Orectolobiformes	GCC30739.1	hypothetical protein chiPu_0009193	LQVLFPSMTLED	SGELSFDSGSICLQISNPDLKD
Chiloscyllium plagiosum	Orectolobiformes	XP_043569882.1	interferon regulatory factor 6-like	LQVLFPSMTLED	SGELSFDSGSICLQISNPDLKD
Carcharodon carcharias	Lamniformes	XP_041059332.1	interferon regulatory factor 6-like	LQVLFPSMTLKD	SGELSFDSTSIRLQISNPDLKD
Pristis pectinata	Rhinopristiformes	XP_051889933.1	interferon regulatory factor 6-like	LQALFPSMTLED	SGELSFDSGSISLQISNPDLKD
Callorhinchus milii	Chimaeriformes	XP_007909076.1	interferon regulatory factor 6	LHDMMPTMSLDE	SGELSFDSGSVRLQISNPDLKD
**[Lobe-finned fish]**					
Protopterus annectens	Ceratodontiformes	XP_043944566.1	interferon regulatory factor 5	LPDMMPKLQIAD	SGEMSWSADSIRLQISNPDLKD
Latimeria chalumnae	Coelacanthiformes	XP_006010437.1	PREDICTED: interferon regulatory factor 5	LPIIMPRLTIAD	SGELAWSADSIRLQISNPDLKD
**[Ray-finned fish]**					
Mylopharyngodon piceus	Cypriniformes	QEE80343.1	interferon regulatory factor 5	LQNFV-KLSIDP	SGELSWSTDSIRLQISNPDLKD
Mastacembelus armatus	Cypriniformes	UNH56802.1	interferon regulatory factor 5	IPNLM-ELSINP	SGELSWSTDSIRLQISNPDVKD
Cyprinus carpio	Cypriniformes	ALC74333.1	interferon regulatory factor 5	LQNFV-KLSIDP	SGELSWSTDSIRLQISNPDLKD
Paralichthys olivaceus	Cypriniformes	AEY55357.1	interferon regulatory factor 5	MPNLM-DLTINP	SGELSWSTDSIRLQISNPDVKD
Takifugu flavidus	Cypriniformes	XP_056878445.1	interferon regulatory factor 5 isoform X1	MPNLM-HLTIGS	SGELNWSTDSVHLRISNPDIKD
Gadus chalcogrammus	Cypriniformes	XP_056434562.1	interferon regulatory factor 5	MPNLD-DLTIDP	SGELSWSTDSIRLQISNPDVKD
Danio aesculapii	Cypriniformes	XP_056311786.1	interferon regulatory factor 5	LQNFV-HLSIDP	SGELSWSTDSIRLQISNPDVKD
Danio rerio	Cypriniformes	NP_001314746.1	interferon regulatory factor 5	LQNFV-HLSIDP	SGELSWSTDSIRLQISNPDLKD
Triplophysa dalaica	Cypriniformes	XP_056603515.1	interferon regulatory factor 5	LTNFI-NLTIDP	SGELSWSTDSIRLQISNTDLKD
Pseudoliparis swirei	Cypriniformes	XP_056280830.1	interferon regulatory factor 5 isoform X1	MPNLM-ELTIQP	SGELSWSTDSIRLQISNPDLKD
Seriola aureovittata	Cypriniformes	XP_056223539.1	interferon regulatory factor 5 isoform X1	MPNLM-DLTINP	SGELSWSTDSIRLQISNPDVKD
Lampris incognitus	Cypriniformes	XP_056131695.1	interferon regulatory factor 5 isoform X1	MPNLR-GLTINP	SGELSWSTDSIRLQISNPDVKD
Rhinichthys klamathensis goyatoka	Cypriniformes	XP_056100435.1	interferon regulatory factor 5	LQNFV-KLSIDP	SGELSWSTDSIRLQISNPDLKD
Betta splendens	Cypriniformes	XP_029018447.1	interferon regulatory factor 5 isoform X1	MPNLM-ELSINP	SGELSWSTDSIRLQISNPDVKD
Misgurnus anguillicaudatus	Cypriniformes	XP_055046568.1	interferon regulatory factor 5	LTNFV-NLTIDP	SGELSWSTDSIRLQISNPDLKD
Periophthalmus magnuspinnatus	Cypriniformes	XP_055087664.1	interferon regulatory factor 5	MPNLM-ELSINP	SGELSWSTDSIRLQISNPDVKD
Boleophthalmus pectinirostris	Cypriniformes	XP_020781960.1	interferon regulatory factor 5 isoform X1	MPNLM-ELSINP	SGELSWSTDSIRLQISNPDVKD
Poeciliopsis prolifica	Cypriniformes	XP_054876779.1	interferon regulatory factor 5 isoform X1	MPDLT-ELSINP	SGELAWSTDSIRLQISNPDVKD
Dunckerocampus dactyliophorus	Cypriniformes	XP_054625125.1	interferon regulatory factor 5	MPNLT-GLAIDS	SGGDSWSIDSIRLQISNPDVKD
Amphiprion fimbria	Cypriniformes	XP_054481073.1	interferon regulatory factor 5	MPNLM-DLTINP	SGELSWSTDSIRLQISNPDVKD
**[Amphibians]**					
Hyla sarda	Anura	XP_056425294.1	interferon regulatory factor 5	IDHMFPALDISD	TGESSWSADSIKLQISHPDLKD
Bombina bombina	Anura	XP_053572316.1	interferon regulatory factor 5 isoform X1	IHNQFPTLQISD	TGESSWSADSIKLQISHPDLKD
Spea bombifrons	Anura	XP_053320861.1	interferon regulatory factor 5	IDHIFPRLGIKD	TGELSWSADSIRLQISHPDLKD
Bufo gargarizans	Anura	XP_044131836.1	interferon regulatory factor 5	IDRIFPMLDIGD	TGESSWSADSIKLQISHPDLKD
Bufo bufo	Anura	XP_040269433.1	interferon regulatory factor 5	IDRMFPMLDIGD	TGESSWSADSIKLQISHPDLKD
Rana temporaria	Anura	XP_040199159.1	interferon regulatory factor 5	INHMFPMLDIQD	SGELSWSADSIKLQISHPDLKD
Xenopus tropicalis	Anura	XP_012814641.2	interferon regulatory factor 5 isoform X1	IGARFPMLHISD	TGESSWSADSIRLQISHPDLKD
Xenopus laevis	Anura	NP_001088065.1	interferon regulatory factor 5 L homeolog	IGARFPMLHISD	TGESSWSADSIRLQISHPDLKD
Nanorana parkeri	Anura	XP_018412577.1	PREDICTED: interferon regulatory factor 5	IDHIFPMLDIQD	SGELSWSADSIKLQISHPDLKD
Geotrypetes seraphini	Gymnophiona	XP_033815605.1	interferon regulatory factor 5	LQDMMPRLHIKA	SGELSWSADSIRLQISHPDLKD
Microcaecilia unicolor	Gymnophiona	XP_030072503.1	interferon regulatory factor 5	LQHMMPRLHIKA	SGELSWSADSIRLQISHPDLKD
Rhinatrema bivittatum	Gymnophiona	XP_029471695.1	interferon regulatory factor 5	LQDMLPSLNINS	SGELSWSADSIRLQISHPDLKD
**[Reptiles (lizards and snakes)]**					
Euleptes europaea	Squamata	XP_056702611.1	interferon regulatory factor 5	LQKIMPALKIAD	SGELSWSADSVRLQISHPDLKD
Eublepharis macularius	Squamata	XP_054845391.1	interferon regulatory factor 5	LQKMMPTLRIAD	SGELSWSADSVHLQISHPDLKD
Podarcis muralis	Squamata	XP_028602706.1	interferon regulatory factor 5	LQKMMPTLQIAD	SGELCWSADSVRLQISHPDLKD
Podarcis raffonei	Squamata	XP_053260676.1	interferon regulatory factor 5	LQKMMPTLQIAD	SGELCWSADSVRLQISHPDLKD
Hemicordylus capensis	Squamata	XP_053110288.1	interferon regulatory factor 5 isoform X1	LQRMMPTLKIAD	SGELSWSADSVRLQISHPDLKD
Sphaerodactylus townsendi	Squamata	XP_048356279.1	LOW QUALITY PROTEIN: interferon regulatory factor 5	LQKMMPTLKIAD	SGELSWSADSVRLQISHPDLKD
Varanus komodoensis	Squamata	XP_044285314.1	interferon regulatory factor 5	LQEMMPTLRIAD	SGELSWSADSVRLQISHPDLKD
Sceloporus undulatus	Squamata	XP_042326862.1	interferon regulatory factor 5	LQKMMPTLRIAD	SGELSWSADSVRLQISHPDLKD
Crotalus tigris	Squamata	XP_039201648.1	interferon regulatory factor 5	LQKMMPTLRIAD	SGELSWSADSVRLQISHPDLKD
Pantherophis guttatus	Squamata	XP_034262996.1	interferon regulatory factor 5	LQKMMPTLRIAD	SGELSWSADSVRLQISHPDLKD
Lacerta agilis	Squamata	XP_033018972.1	interferon regulatory factor 5	LQKMMPTLQIAD	SGELCWSADSVRLQISHPDLKD
Thamnophis elegans	Squamata	XP_032094692.1	LOW QUALITY PROTEIN: interferon regulatory factor 5	LQKMMPTLRIAD	SGELSWSADSVRLQISHPDLKD
Protobothrops mucrosquamatus	Squamata	XP_015681330.1	interferon regulatory factor 5	LQKMMPTLRIAD	SGELSWSADSVRLQISHPDLKD
Pseudonaja textilis	Squamata	XP_026578475.1	interferon regulatory factor 5	LQKMMPTLRIAD	SGELSWSADSVRLQISHPDLKD
Python bivittatus	Squamata	XP_007444867.1	interferon regulatory factor 5 isoform X1	LQKMMPTLRIAD	SGELSWSADSVRLQISHPDLKD
Pogona vitticeps	Squamata	XP_020668112.1	interferon regulatory factor 5 isoform X2	LQKMLPTLRIAD	SGELSWSADSVRLQISHPDLKD
Agkistrodon contortrix contortrix	Squamata	JAV50245.1	interferon regulatory factor 5	LQKMMPTLRIAD	SGELSWSADSVRLQISHPDLKD
**[Reptiles (turtles)]**					
Malaclemys terrapin pileata	Testudines	XP_053866586.1	interferon regulatory factor 5	LQKMTTLTIAD-	SGELSWSADSIRLQISHPDLKD
Caretta caretta	Testudines	XP_048692757.1	interferon regulatory factor 5 isoform X1	LQKMTTLTIVD-	SGELSWSADSIRLQISHPDLKD
Mauremys mutica	Testudines	XP_44847579.1	interferon regulatory factor 5	LQKLTTLTIAD-	SGELSWSADSIRLQISHPDLKD
Mauremys reevesii	Testudines	XP_039375931.1	interferon regulatory factor 5	LQKLTTLTIAD-	SGELSWSADSIRLQISHPDLKD
Chelonia mydas	Testudines	XP_043377367.1	interferon regulatory factor 5	LQKMTTLTIAD-	SGELSWSADSIRLQISHPDLKD
Dermochelys coriacea	Testudines	XP_043355636.1	interferon regulatory factor 5 isoform X1	LQKMTTLTIAD-	SGELSWSADSIRLQISHPDLKD
Chrysemys picta bellii	Testudines	XP_008172295.1	interferon regulatory factor 5	LQKMTTLTIAD-	SGELSWSADSIRLQISHPDLKD
Trachemys scripta elegans	Testudines	XP_034619617.1	interferon regulatory factor 5	LQKMTTLTIAD-	SGELSWSADSIRLQISHPDLKD
Chelonoidis abingdonii	Testudines	XP_032640633.1	interferon regulatory factor 5	LQKLTTLTIAD-	SGELSWSADSIRLQISHPDLKD
Gopherus evgoodei	Testudines	XP_030439453.1	interferon regulatory factor 5	LQKLTTLTIAD-	SGELSWSADSIRLQISHPDLKD
Gopherus flavomarginatus	Testudines	XP_050785942.1	interferon regulatory factor 5	LQKLTTLTIAD-	SGELSWSADSIRLQISHPDLKD
Terrapene carolina triunguis	Testudines	XP_026516090.1	interferon regulatory factor 5	LQKMTTLTIAD-	SGELSWSADSIRLQISHPDLKD

**[Reptiles (crocodilians)]**						
	Alligator sinensis	Crocodilia	XP_006038017.1	interferon regulatory factor 5	LQKMTALTIAVT	SGELSWSADSIRLQISHPDLKD
	Alligator mississippiensis	Crocodilia	KYO31907.1	interferon regulatory factor 5	LQKMTALTIADS	SGELSWSADSIRLQISHPDLKD
	Gavialis gangeticus	Crocodilia	XP_019365234.1	PREDICTED: interferon regulatory factor 5	---MTALTIADP	SGELSWSADSIRLQISHPDLKD
	Crocodylus porosus	Crocodilia	XP_019399375.1	PREDICTED: interferon regulatory factor 5	LQKMTTLTIADP	SGELSWSADSIRLQISHPDLKD
**[Aves]**						
	Apteryx rowi	Apterygiformes	XP_025946890.1	interferon regulatory factor 5	LQKMMSLSIEVT	SGELSWSADSIPLQISHPDLKD
	Nothoprocta perdicaria	Tinamiformes	XP_025900121.1	interferon regulatory factor 5	LQELTSLSIAEV	SGELSWSADSVPLHISPPDLED
	Tympanuchus pallidicinctus	Galliformes	XP_052537058.1	interferon regulatory factor 5	LQKLMSLSIDDC	SGELSWSADSVPLQISQPDLKD
	Lagopus muta	Galliformes	XP_048787176.1	LOW QUALITY PROTEIN: interferon regulatory factor 5	LQKLMSLSIDDC	SGELSWSADSVPLQISQPDLKD
	Lagopus leucura	Galliformes	XP_042746983.1	interferon regulatory factor 5	LQKLMSLSIDDC	SGELSWSADSVPLQISQPDLKD
	Gallus gallus	Galliformes	NP_001026758.2	interferon regulatory factor 5	LQKLMSLSIDDC	SGELSWSADSVPLQISQPDLKD
	Coturnix japonica	Galliformes	XP_015709923.1	interferon regulatory factor 5	LQKLMSLSINDC	SGELSWSADSVPLQISQPDLKD
	Meleagris gallopavo	Galliformes	XP_010705025.1	interferon regulatory factor 5	LQKLMSLSIDDC	SGELSWSADSVPLQISQPDLKD
	Phasianus colchicus	Galliformes	XP_031460923.1	interferon regulatory factor 5	LQKLTSLSIKDC	SGELSWSADSVPLQISQPDLKD
	Numida meleagris	Galliformes	XP_021239885.1	interferon regulatory factor 5 isoform X1	LQQLMSLSIDDC	SGELSWSADSVPLQISQPDLKD
	Centrocercus urophasianus	Galliformes	XP_042665190.1	interferon regulatory factor 5 isoform X1	LQKLMSLSIDDC	
	Cygnus atratus	Anseriformes	XP_035427142.1	interferon regulatory factor 5 isoform X1	LQKLMSLSIDGE	SGELSWSADSIPLQISHPDLKD
	Cygnus olor	Anseriformes	XP_040401218.1	interferon regulatory factor 5	LQKLMSLSIDGE	SGELSWSADSIPLQISHPDLKD
	Aythya fuligula	Anseriformes	XP_032057735.1	interferon regulatory factor 5		SGELSWSADSIPLQISHPDLKD
	Cuculus canorus	Cuculiformes	XP_053929629.1	LOW QUALITY PROTEIN: interferon regulatory factor 5	LQKMTSLSISDT	SGELSWSADSIPLQISHPDLKD
	Columba livia	Columbiformes	PKK22067.1	interferon regulatory factor 5		SGELSWSADSVPLHISHPDLKD
	Calidris pugnax	Charadriiformes	XP_014816226.1	PREDICTED: interferon regulatory factor 5		SGELSWSADSIPLQISHPDLKD
	Rissa tridactyla	Charadriiformes	XP_054044629.1	interferon regulatory factor 5	LQKMTSLSIDDT	SGELSWSADSIPLQISHPDLKD
	Charadrius vociferus	Charadriiformes	XP_009881341.1	PREDICTED: interferon regulatory factor 5		
	Grus americana	Gruiformes	XP_054686865.1	interferon regulatory factor 5 isoform X1	LQKMTSLSISDT	SGELSWSADSIPLQISHPDLKD
	Nipponia nippon	Pelecaniformes	XP_009460900.1	PREDICTED: interferon regulatory factor 5		WCELSWSADSIPLQISHPDLKD
	Accipiter gentilis	Accipitriformes	XP_049669677.1	interferon regulatory factor 5	LQKMMSLSINDT	SGELSWSADSIPLQISHPDLKD
	Harpia harpyja	Accipitriformes	XP_052644837.1	LOW QUALITY PROTEIN: interferon regulatory factor 5	LQKMTSLSINDT	SGELSWSADSIPLQISHPDLKD
	Aquila chrysaetos chrysaetos	Accipitriformes	XP_040979479.1	LOW QUALITY PROTEIN: interferon regulatory factor 5	LQKMTSLSINDT	SGELSWSADSIPLQISHPDLKD
	Haliaeetus leucocephalus	Accipitriformes	XP_010567096.1	PREDICTED: interferon regulatory factor 5		SGELSWSADSIPLQISHPDLKD
	Gymnogyps californianus	Cathartiformes	XP_050757328.1	LOW QUALITY PROTEIN: interferon regulatory factor 5	LQKMTSLSIDDT	SGELSWSADSIPLQISHPDLKD
	Indicator indicator	Piciformes	XP_054255730.1	interferon regulatory factor 5	LQKMTSLSINDT	SGELSWSADSIPLQISHPDLKD
	Dryobates pubescens	Piciformes	XP_054033953.1	interferon regulatory factor 5	LQKMTSLSIIDT	SGELSWSADSIPLQISHPDLKD
	Falco biarmicus	Falconiformes	XP_056196048.1	interferon regulatory factor 5 isoform X1	LQKMTSLSIKDT	SGELSWSADSIPLQISHPDLKD
	Falco peregrinus	Falconiformes	XP_055663756.1	interferon regulatory factor 5 isoform X1	LQKMTSLSIKDT	SGELSWSADSIPLQISHPDLKD
	Falco cherrug	Falconiformes	XP_055567267.1	interferon regulatory factor 5 isoform X1	LQKMTSLSIKDT	SGELSWSADSIPLQISHPDLKD
	Falco naumanni	Falconiformes	XP_040451098.1	interferon regulatory factor 5	LQKMTSLSIKDT	SGELSWSADSIPLQISHPDLKD
	Falco rusticolus	Falconiformes	XP_037244690.1	LOW QUALITY PROTEIN: interferon regulatory factor 5	LQKMTSLSIKDT	SGELSWSADSIPLQISHPDLKD
	Strigops habroptila	Psittaciformes	XP_030334001.1	interferon regulatory factor 5	LQMTSLSINDTH	SGELSWSADSIPLHISHPDLKD
	Oenanthe melanoleuca	Passeriformes	XP_056338052.1	interferon regulatory factor 5	LQKMTSLSIDDT	SGELSWSADSIPLQISHPDLKD
	Agelaius phoeniceus	Passeriformes	XP_054487248.1	interferon regulatory factor 5	LHKMTSLSIDDT	SGELSWSADSIPLQISHPDLKD
	Molothrus ater	Passeriformes	XP_036256463.1	interferon regulatory factor 5	LHEMTSLSIDDT	SGELSWSADSIPLQISHPDLKD
	Melozone crissalis	Passeriformes	XP_054150075.1	interferon regulatory factor 5	LHKMTSLSIDDS	SGELSWSADSIPLQISHPDLKD
	Vidua macroura	Passeriformes	XP_053833058.1	interferon regulatory factor 5	LHKMTSLSIDDT	SGELSWSADSIPLQISHPDLKD
	Vidua chalybeata	Passeriformes	XP_053798403.1	interferon regulatory factor 5	LHKMTSLSIDDT	SGELSWSADSIPLQISHPDLKD
	Serinus canaria	Passeriformes	XP_050825810.1	interferon regulatory factor 5 isoform X1	LHKMTSLSIDDT	SGELSWSADSIPLQISHPDLKD
	Manacus candei	Passeriformes	XP_051629936.1	interferon regulatory factor 5	LQKMTALSISDT	SGELSWSADSIPLQISHPDLKD
	Pipra filicauda	Passeriformes	XP_027576179.1	interferon regulatory factor 5 isoform X1	LQKMTALSIDDT	SGELSWSADSIPLQISHPDLKD
	Chiroxiphia lanceolata	Passeriformes	XP_032543997.1	interferon regulatory factor 5 isoform X1	LQKMTALSINDT	SGELSWSADSIPLQISHPDLKD
	Corapipo altera	Passeriformes	XP_027525533.1	LOW QUALITY PROTEIN: interferon regulatory factor 5	LQKMTALSINDT	SGELSWSADSIPLQISHPDLKD
	Neopelma chrysocephalum	Passeriformes	XP_027557358.1	interferon regulatory factor 5 isoform X1	LQKMTALSIDDT	SGELSWSADSIPLQISHPDLKD
	Lepidothrix coronata	Passeriformes	XP_017694385.1	PREDICTED: interferon regulatory factor 5	LQKMTALSIDDT	SGELSWSADSIPLQISHPDLKD
	Myiozetetes cayanensis	Passeriformes	XP_050180706.1	interferon regulatory factor 5	LQKMTALSIDDT	SGELSWSADSIPLQISHPDLKD
	Corvus hawaiiensis	Passeriformes	XP_048157547.1	interferon regulatory factor 5	LQKMTSLSIDDT	SGELSWSADSIPLQISHPDLKD
	Corvus moneduloides	Passeriformes	XP_031962385.1	interferon regulatory factor 5 isoform X1	LQKMTSLSIDDT	SGELSWSADSIPLQISHPDLKD
	Corvus cornix	Passeriformes	XP_039426951.1	interferon regulatory factor 5 isoform X1	LQKMTSLSIDDT	SGELSWSADSIPLQISHPDLKD
	Corvus brachyrhynchos	Passeriformes	XP_017594875.1	PREDICTED: LOW QUALITY PROTEIN: interferon regulatory factor 5	LQKMTSLSINGE	SGELSWSADSIPLQISHPDLKD
	Catharus ustulatus	Passeriformes	XP_032914275.1	interferon regulatory factor 5	LQKMTSLSIDDT	SGELSWSADSIPLQISHPDLKD
	Hirundo rustica	Passeriformes	XP_039917353.1	interferon regulatory factor 5	LHKMTSLSIDDT	SGELSWSADSIPLQISHPDLKD
	Parus major	Passeriformes	XP_015471466.1	interferon regulatory factor 5	LNMTSLSIDDTQ	SGELSWSADSIPLQISHPDLKD
	Pseudopodoces humilis	Passeriformes	XP_014117409.1	PREDICTED: interferon regulatory factor 5 isoform X1	LNMTSLSIDDTQ	SGELSWSADSIPLHISHPDLKD
	Lonchura striata domestica	Passeriformes	XP_021402639.1	interferon regulatory factor 5	LHKMTSLSIDDK	SGELSWSADSIPLQISHPDLKD
	Taeniopygia guttata	Passeriformes	XP_030117532.3	interferon regulatory factor 5	LHKMTSLSIDDT	SGELSWSADSIPLQISHPDLKD
	Camarhynchus parvulus	Passeriformes	XP_030816741.1	interferon regulatory factor 5	LHKMTSLSIDDT	SGELSWSADSIPLQISHPDLKD
	Sturnus vulgaris	Passeriformes	XP_014746235.1	PREDICTED: interferon regulatory factor 5	LQKMTSLSIDDT	SGELSWSADSIPLQISHPDLKD
	Pyrgilauda ruficollis	Passeriformes	XP_041326175.1	interferon regulatory factor 5 isoform X1	LHKMTSLSIHDT	
	Onychostruthus taczanowskii	Passeriformes	XP_041262999.1	interferon regulatory factor 5 isoform X1	LHKMTSLSIDDT	
	Passer montanus	Passeriformes	XP_039560480.1	interferon regulatory factor 5		SGELSWSADSIPLQISHPDLKD
	Motacilla alba	Passeriformes	XP_038010570.1	interferon regulatory factor 5 isoform X1	LHKMTSLSIDDT	SGELSWSADSIPLQISHPDLKD
	Ficedula albicollis	Passeriformes	XP_005061576.1	PREDICTED: interferon regulatory factor 5		SGELSWSADSIPLQISHPDQVR
	Willisornis vidua	Passeriformes	KAJ7421607.1	interferon regulatory factor 5		SGELSWSADSIPLQISHPDLKD
**[Mammals (monotremes)]**						
	Ornithorhynchus anatinus	Monotremata	XP_001509633.3	interferon regulatory factor 5	LQRMLPTLSITD	SGELSWSADSIRLQISNPDLKD
	Tachyglossus aculeatus	Monotremata	XP_038608369.1	interferon regulatory factor 5	LQQMLPTLSITD	SGELSWSADSIRLQISNPDLKD
**[Mammals (marsupials)]**						
	Monodelphis domestica	Didelphimorphia	XP_056656636.1	interferon regulatory factor 5	LQRMLPSLRITG	SGELSWSADSIRLQISNPDLKD
	Gracilinanus agilis	Didelphimorphia	XP_044534957.1	interferon regulatory factor 5	LQRMLPSLRITG	SGELSWSADSIRLQISNPDLKD
	Trichosurus vulpecula	Diprotodontia	XP_036614471.1	interferon regulatory factor 5 isoform X1	LQRMLPTLRLTE	SGELSWSADSIRLQISNPDLKD
	Vombatus ursinus	Diprotodontia	XP_027695980.1	interferon regulatory factor 5 isoform X1	LQRMLPTLSITE	SGELSWSADSIRLQISNPDLKD
	Phascolarctos cinereus	Diprotodontia	XP_020857998.1	interferon regulatory factor 5 isoform X1	LQRMLPSLSITE	SGELSWSADSIRLQISNPDLKD
	Sarcophilus harrisii	Dasyuromorphia	XP_023360627.1	interferon regulatory factor 5	LQKMLPTLRLTE	SGELSWSADSIRLQISNPDLKD
	Dromiciops gliroides	Microbiotheria	XP_043822512.1	interferon regulatory factor 5	LQKILPSLRITE	SGELSWSADSIRLQISNPDLKD

**[Mammals (moles, shrews, hedgehogs)]**						
	Galemys pyrenaicus	Eulipotyphla	KAG8524502.1	Interferon regulatory factor 5	EEELCPLLSSTE	SGELSWSADSIRLQISNPDLKD
	Talpa occidentalis	Eulipotyphla	XP_037368872.1	Interferon regulatory factor 5	LQRMLPSLSLTE	SGELSWSADSIRLQISNPDLKD
	Erinaceus europaeus	Eulipotyphla	XP_016045128.1	PREDICTED: interferon regulatory factor 5	LQRMLPSLKLTG	SGELSWSADSIRLQISNPDLKD
	Condylura cristata	Eulipotyphla	XP_004677083.1	PREDICTED: interferon regulatory factor 5	LQRMLPSLSLTE	SGELSWSADSIRLQISNPDLKD
**[Mammals (rodents)]**						
	Rattus norvegicus	Rodentia	NP_001100056.1	interferon regulatory factor 5	LQRMLPGLSITE	SGELSWSADSIRLQISNPDLKD
	Rattus rattus	Rodentia	XP_032762721.1	interferon regulatory factor 5 isoform X1	LQRMLPGLSITE	SGELSWSADSIRLQISNPDLKD
	Mus musculus	Rodentia	NP_001239311.1	interferon regulatory factor 5 isoform 1	LQRMLPGLSITE	SGELSWSADSIRLQISNPDLKD
	Mus pahari	Rodentia	XP_021047048.1	interferon regulatory factor 5 isoform X1	LQKMLPGLRLTE	SGELSWSADSIRLQISNPDLKD
	Mus caroli	Rodentia	XP_029334231.1	interferon regulatory factor 5 isoform X1	LQRMLPGLSITD	SGELSWSADSIRLQISNPDLKD
	Peromyscus leucopus	Rodentia	XP_028722043.1	interferon regulatory factor 5 isoform X1	LQRMLPSLTITD	SGELSWSADSIRLQISNPDLKD
	Peromyscus maniculatus bairdii	Rodentia	XP_015853547.1	interferon regulatory factor 5	LQRMLPSLTITD	SGELSWSADSIRLQISNPDLKD
	Onychomys torridus	Rodentia	XP_036036110.1	interferon regulatory factor 5 isoform X1	LQRMLPSLSITD	SGELSWSADSIRLQISNPDLKD
	Marmota monax	Rodentia	KAF7470788.1	interferon regulatory factor 5	LQRMLPGLSITE	SGELSWSADSIRLQISNPDLKD
	Marmota marmota marmota	Rodentia	XP_015349330.1	interferon regulatory factor 5 isoform X1	LQRMLPGLSITE	SGELSWSADSIRLQISNPDLKD
	Marmota flaviventris	Rodentia	XP_027811613.1	interferon regulatory factor 5	LQRMLPGLSITE	SGELSWSADSIRLQISNPDLKD
	Microtus oregoni	Rodentia	XP_041531338.1	interferon regulatory factor 5	LQRMLPGLSITE	SGELSWSADSIRLQISNPDLKD
	Microtus ochrogaster	Rodentia	XP_026644339.1	interferon regulatory factor 5 isoform X1	LQRMLPGLSITE	SGELSWSADSIRLQISNPDLKD
	Arvicola amphibius	Rodentia	XP_038173944.1	interferon regulatory factor 5 isoform X1	LQRMLPGLSITE	SGELSWSADSIRLQISNPDLKD
	Ictidomys tridecemlineatus	Rodentia	KAG3277510.1	interferon regulatory factor 5	LQRMLPGLSITE	SGELSWSADSIRLQISNPDLKD
	Cricetulus griseus	Rodentia	XP_035297474.1	interferon regulatory factor 5 isoform X1	LQRMLPGLSITE	SGELSWSADSIRLQISNPDLKD
	Arvicanthis niloticus	Rodentia	XP_034374754.1	interferon regulatory factor 5 isoform X1	LQRMLPGLSITE	SGELSWSADSIRLQISNPDLKD
	Fukomys damarensi	Rodentia	XP_010631767.1	interferon regulatory factor 5	LQKMLPGLRLTE	SGEFSWSADSIRLQISNPDLKD
	Mastomys coucha	Rodentia	XP_031237236.1	interferon regulatory factor 5	LQKMLPGLRLTE	SGELSWSADSIRLQISNPDLKD
	Nannospalax galili	Rodentia	XP_029425901.1	interferon regulatory factor 5	LQKMLPGLRLTE	SGELSWSADSIRLQISNPDLKD
	Grammomys surdaster	Rodentia	XP_028618441.1	interferon regulatory factor 5 isoform X1	LQKMLPGLSITE	SGELSWSADSIRLQISNPDLKD
	Urocitellus parryii	Rodentia	XP_026248560.1	interferon regulatory factor 5 isoform X1	LQRMLPGLSITE	SGELSWSADSIRLQISNPDLKD
	Octodon degus	Rodentia	XP_023558657.1	interferon regulatory factor 5 isoform X1	LQRMLPGLSLTE	SGELSWSVDSIRLQISNPDLKD
	Cavia porcellus	Rodentia	XP_003475230.2	interferon regulatory factor 5 isoform X1	LQKMLPGLSLTE	SGELSWSADSIRLQISNPDLKD
	Meriones unguiculatus	Rodentia	XP_021496962.1	interferon regulatory factor 5 isoform X1	LQRMLPGLSITE	AGELSWSADSIRLQISNPDLKD
	Heterocephalus glaber	Rodentia	XP_021093196.1	interferon regulatory factor 5	LQKMLPELRLTE	SGEFSWSADSIRLQISNPDLKD
	Castor canadensis	Rodentia	XP_020015948.1	LOW QUALITY PROTEIN: interferon regulatory factor 5	LQRMLPGLSITE	SGELSWSADSIRLQISNPDLKD
	Dipodomys ordii	Rodentia	XP_012875449.1	PREDICTED: interferon regulatory factor 5	LQRMLPSLNITE	SGELSWSADSIRLQISNPDLKD
	Dipodomys spectabilis	Rodentia	XP_042552043.1	interferon regulatory factor 5 isoform X1	LQRMLPSLNITE	SGELSWSADSIRLQISNPDLKD
	Heterocephalus glaber	Rodentia	EHB08653.1	interferon regulatory factor 5	EEKMLPELRLTE	SGEFSWSADSIRLQISNPDLKD
	Mesocricetus auratus	Rodentia	XP_040584616.1	interferon regulatory factor 5	LQRMLPGLSITE	SGELSWSADSIRLQISNPDLKD
**[Mammals (bats)]**						
	Pteropus giganteus	Chiroptera	XP_039716880.1	interferon regulatory factor 5	LQRMLPSLSLTE	SGELSWSADSIRLQISNPDLKD
	Molossus molossus	Chiroptera	XP_036103098.1	interferon regulatory factor 5	LQRMLPSLSLTE	SGELSWSADSIRLQISNPDLKD
	Rousettus aegyptiacus	Chiroptera	XP_016004160.2	interferon regulatory factor 5 isoform X1	LQRMLPSLSLTE	SGELSWSADSIRLQISNPDLKD
	Phyllostomus discolor	Chiroptera	XP_035866782.1	interferon regulatory factor 5 isoform X1	LQRMLPSLSLTE	SGELSWSADSIRLQISNPDLKD
	Rousettus aegyptiacus	Chiroptera	KAF6418582.1	interferon regulatory factor 5	LQRMLPSLSLTE	SGELSWSADSIRLQISNPDLKD
	Pipistrellus kuhlii	Chiroptera	KAF6338147.1	interferon regulatory factor 5	LQRMLPSLSLTE	SGELSWSADSIRLQISNPDLKD
	Myotis myotis	Chiroptera	KAF6319617.1	interferon regulatory factor 5	LQRMLPSLSLTE	SGELSWSADSIRLQISNPDLKD
	Rhinolophus ferrumequinum	Chiroptera	KAF6276025.1	interferon regulatory factor 5	LQRMLPSLSITE	SGELSWSADSIRLQISNPDLKD
	Phyllostomus discolor	Chiroptera	KAF6086051.1	interferon regulatory factor 5	LQRMLPSLSLTE	SGELSWSADSIRLQISNPDLKD
	Rhinolophus ferrumequinum	Chiroptera	XP_032955178.1	interferon regulatory factor 5 isoform X1	LQRMLPSLSITE	SGELSWSADSIRLQISNPDLKD
	Sturnira hondurensis	Chiroptera	XP_036905129.1	interferon regulatory factor 5 isoform X1	LQRMLPSLSLTE	SGELSWSADSIRLQISNPDLKD
	Pteropus alecto	Chiroptera	XP_006910593.2	interferon regulatory factor 5 isoform X1	LQRMLPSLSLTE	SGELSWSADSIRLQISNPDLKD
	Myotis lucifugus	Chiroptera	XP_006088437.2	interferon regulatory factor 5 isoform X1	LQRMLPGLSLTE	SGELSWSADSIRLQISNPDLKD
	Pteropus vampyrus	Chiroptera	XP_023384691.1	interferon regulatory factor 5 isoform X1	LQRMLPSLSLTE	SGELSWSADSIRLQISNPDLKD
	Miniopterus natalensis	Chiroptera	XP_016068803.1	PREDICTED: interferon regulatory factor 5	LQRMLPSLSLTE	SGELSWSADSIRLQISNPDLKD
	Myotis davidii	Chiroptera	ELK24456.1	Interferon regulatory factor 5	LQRMLPGLSLTE	SGELSWSADSIRLQISNPDLKD
	Pteropus alecto	Chiroptera	ELK13867.1	Interferon regulatory factor 5	LQRMLPSLSLTE	SGELSWSADSIRLQISNPDLKD
	Rhinolophus sinicus	Chiroptera	XP_019607113.1	PREDICTED: interferon regulatory factor 5 isoform X1	LQRMLPSLSITE	SGELSWSADSIRLQISNPDLKD
**[Mammals (primates)]**						
	Theropithecus gelada	Primates	XP_025234073.1	interferon regulatory factor 5 isoform X1	LQRMLPSLSLTD	SGELSWSADSIRLQISNPDLKD
	Carlito syrichta	Primates	XP_008061786.1	interferon regulatory factor 5 isoform X1	LQRMLPSLSITE	SGELSWSADSIRLQISNPDLKD
	Aotus nancymaae	Primates	XP_021529320.1	interferon regulatory factor 5	LQRMLPSLSLTD	SGELSWSADSIRLQISNPDLKD
	Microcebus murinus	Primates	XP_012644631.2	interferon regulatory factor 5 isoform X1	LQRMLPNLSITE	SGELSWSADSIRLQISNPDLKD
	Propithecus coquereli	Primates	XP_012520246.1	PREDICTED: interferon regulatory factor 5	LQRMLPNLSITE	SGELSWSADSIRLQISNPDLKD
	Cercocebus atys	Primates	XP_011943404.1	PREDICTED: interferon regulatory factor 5	LQRMLPSLSLTD	SGELSWSADSIRLQISNPDLKD
	Mandrillus leucophaeus	Primates	XP_011851279.1	PREDICTED: interferon regulatory factor 5 isoform X1	LQRMLPSLSLTD	SGELSWSADSIRLQISNPDLKD
	Colobus angolensis palliatus	Primates	XP_011794962.1	PREDICTED: interferon regulatory factor 5 isoform X1	LQRMLPSLSLTD	SGELSWSADSIRLQISNPDLKD
	Callithrix jacchus	Primates	XP_002752074.1	interferon regulatory factor 5 isoform X2	LQRMLPSLSLTV	SGELSWSADSIRLQISNPDLKD
	Macaca fascicularis	Primates	XP_045244326.1	interferon regulatory factor 5	LQRMLPSLSLTD	SGELSWSADSIRLQISNPDLKD
	Macaca mulatta	Primates	XP_028702109.1	interferon regulatory factor 5 isoform X1	LQRMLPSLSLTD	SGELSWSADSIRLQISNPDLKD
	Trachypithecus francoisi	Primates	XP_033049797.1	interferon regulatory factor 5 isoform X2	LQRMLPSLSLTD	SGELSWSADSIRLQISNPDLKD
	Hylobates moloch	Primates	XP_032617570.1	interferon regulatory factor 5 isoform X1	LQRMLPSLSLTD	SGELSWSADSIRLQISNPDLKD
	Sapajus apella	Primates	XP_032128364.1	interferon regulatory factor 5 isoform X1	LQRMLPSLSLTD	SGELSWSADSIRLQISNPDLKD
	Piliocolobus tephrosceles	Primates	XP_031791931.1	interferon regulatory factor 5	LQRMLPSLSLTD	SGELSWSADSIRLQISNPDLKD
	Papio anubis	Primates	XP_009202106.1	interferon regulatory factor 5 isoform X1	LQRMLPSLSLTD	SGELSWSADSIRLQISNPDLKD
	Rhinopithecus roxellana	Primates	XP_030789133.1	interferon regulatory factor 5 isoform X1	LQRMLPSLSLTD	SGELSWSADSIRLQISNPDLKD
	Nomascus leucogenys	Primates	XP_030682359.1	interferon regulatory factor 5 isoform X1	LQKMLPSLSLTD	SGELSWSADSIRLQISNPDLKD
	Cebus imitator	Primates	XP_017405019.1	interferon regulatory factor 5 isoform X1	LQRMLPSLSLTD	SGELSWSADSIRLQISNPDLKD
	Chlorocebus sabaeus	Primates	XP_007981057.1	interferon regulatory factor 5 isoform X1	LQRMLPSLSLTD	SGELSWSADSIRLQISNPDLKD
	Saimiri boliviensis boliviensis	Primates	XP_039328942.1	interferon regulatory factor 5 isoform X1	LQRMLPSLSLTD	SGELSWSADSIRLQISNPDLKD
	Gorilla gorilla gorilla	Primates	XP_004046244.1	interferon regulatory factor 5 isoform X4	LQRMLPSLSLTV	SGELSWSADSIRLQISNPDLKD
	Pongo abelii	Primates	XP_024105566.1	nterferon regulatory factor 5 isoform X1	LQRMLPSLSLTD	SGELSWSADSIRLQISNPDLKD
	Pan troglodytes	Primates	PNI99430.1	IRF5 isoform 5	LQRMLPSLSLTV	SGELSWSADSIRLQISNPDLKD
	Homo sapiens	Primates	AAA96056.1	interferon regulatory factor 5	LQRMLPSLSLTD	SGELSWSADSIRLQISNPDLKD

*Turtles and bony fish BOTH have one "missing" amino acid in their NES sequence

Table S3. STAT protein sequences used for Figure 3A.[a]

[a]STAT family classification is based on the phylogenetic placement. Sequences with ambiguous or incorrect annotations are shown with yellow background.

			STAT1		STAT2	
**Species**	**Common name**	**Order (subgroup)**	**Accession #**	**Description**	**Accession #**	**Description**
**[Cartilaginous fish]**						
Callorhinchus milii	Australian ghostshark	Chimaeriformes	XP_042192024.1	signal transducer and activator of transcription 1-alpha/beta	XP_042200176.1	signal transducer and activator of transcription 1-alpha/beta-like isoform X1
Stegostoma tigrinum	Zebra shark	Orectolobiformes	XP_048390215.1	signal transducer and activator of transcription 1-alpha/beta-like	XP_059499440.1	signal transducer and activator of transcription 1-like isoform X1
**[Ray-finned fish]**						
Polypterus senegalus	Senegal bichir	Polypteriformes	XP_039599311.1	signal transducer and activator of transcription 1-alpha/beta-like	XP_039602529.1	signal transducer and activator of transcription 2
**[Eutherians]**						
Homo sapiens	Human	Primate (great apes)	NP_009330.1	signal transducer and activator of transcription 1-alpha/beta isoform alpha	NP_005410.1	signal transducer and activator of transcription 2 isoform 1

			STAT3		STAT4	
**Species**	**Common name**	**Order (subgroup)**	**Accession #**	**Description**	**Accession #**	**Description**
**[Cartilaginous fish]**						
Callorhinchus milii	Australian ghostshark	Chimaeriformes	XP_042199113.1	signal transducer and activator of transcription 3	XP_007888353.1	signal transducer and activator of transcription 4
Stegostoma tigrinum	Zebra shark	Orectolobiformes	XP_048416234.1	signal transducer and activator of transcription 3	XP_048390892.1	signal transducer and activator of transcription 4
**[Ray-finned fish]**						
Polypterus senegalus	Senegal bichir	Polypteriformes	XP_039596077.1	signal transducer and activator of transcription 3 isoform X3	XP_039599306.1	signal transducer and activator of transcription 4 isoform X1
**[Eutherians]**						
Homo sapiens	Human	Primate (great apes)	NP_644805.1	signal transducer and activator of transcription 3 isoform 1	AAH31212.1	STAT4 protein

			STAT5		STAT6	
**Species**	**Common name**	**Order (subgroup)**	**Accession #**	**Description**	**Accession #**	**Description**
**[Cartilaginous fish]**						
Callorhinchus milii	Australian ghostshark	Chimaeriformes	XP_042199109.1	signal transducer and activator of transcription 5A		
Stegostoma tigrinum	Zebra shark	Orectolobiformes	XP_048416231.1	signal transducer and activator of transcription 5A	XP_059499437.1	signal transducer and activator of transcription 5B-like isoform X1
**[Ray-finned fish]**						
Polypterus senegalus	Senegal bichir	Polypteriformes	XP_039596639.1	signal transducer and activator of transcription 5A	XP_039603197.1	signal transducer and activator of transcription 6 isoform X2
**[Eutherians]**						
Homo sapiens	Human	Primate (great apes)	AAB06589.1	Stat5A	NP_001171550.1	signal transducer and activator of transcription 6 isoform 1
			NP_036580.2	signal transducer and activator of transcription 5B		

Species	Common name	Order (subgroup)	Accession #	Description
**[Jawless fish]**				
Petromyzon marinus	Sea lamprey	Petromyzontiformes	XP_032802147.1	signal transducer and activator of transcription 1-alpha/beta
			XP_032814978.1	signal transducer and activator of transcription 1-alpha/beta-like
			XP_032815009.1	signal transducer and activator of transcription 5A-like
			XP_032813201.1	signal transducer and activator of transcription 5B-like

Table S4. Signature motifs found in avian IRF5 and IRF6 proteins.*

*The avian species are ordered roughly following the avian classification shown in Stiller et al. (2024) Nature 629: 851-860. The accession numbers for IRF5 sequences are found in Supplemental Table S2.

Species	Family	Order	IRF5 signature	IRF6 signature	Acc #
**[Infraclass: Palaeognathae]**					
Apteryx rowi	Apterygidae	Apterygiformes	YDG	YDG	XP_025933003.1
Nothoprocta perdicaria	Tinamidae	Tinamiformes	YDG	YDG	XP_025891913.1
**[Infraclass:Neognathae]**					
**[Superorder: Galloanserae]**					
Tympanuchus pallidicinctus	Phasianidae	Galliformes	YDG	YDG	XP_052554087.1
Lagopus muta	Phasianidae	Galliformes	YDG	YDG	XP_048781927.1
Lagopus leucura	Phasianidae	Galliformes	YDG	YDG	XP_042740375.1
Gallus gallus	Phasianidae	Galliformes	YDG	YDG	XP_046789012.1
Coturnix japonica	Phasianidae	Galliformes	YDG	YDG	XP_015740711.1
Meleagris gallopavo	Phasianidae	Galliformes	YDG	YDG	XP_003213004.1
Phasianus colchicus	Phasianidae	Galliformes	YDG	YDG	XP_031461661.1
Numida meleagris	Numididae	Galliformes	YDG	YDG	XP_021232906.1
Cygnus atratus	Anatidae	Anseriformes	YDG	YDG	XP_035417452.1
Cygnus olor	Anatidae	Anseriformes	YDG	YDG	XP_040392004.1
Aythya fuligula	Anatidae	Anseriformes	YDG	YDG	XP_032059149.1
**[Clade: Columbaves]**					
Cuculus canorus	Cuculidae	Cuculiformes	LDG	YDG	XP_053943496.1
Columba livia	Columbidae	Columbiformes	FDG	YDG	KAK2521884.1
**[Clade: Cursorimorphae]**					
Calidris pugnax	Scolopacidae	Charadriiformes	FDG	YDG	XP_014803913.1
Rissa tridactyla	Laridae	Charadriiformes	FDG	YDG	XP_054037083.1
Grus americana	Gruidae	Gruiformes	FDG	YDG	XP_054660137.1
**[Clade: Afroaves]**					
Accipiter gentilis	Accipitridae	Accipitriformes	FDG	YDG	XP_049688944.1
Harpia harpyja	Accipitridae	Accipitriformes	FDG	YDG	XP_052671427.1
Aquila chrysaetos chrysaetos	Accipitridae	Accipitriformes	FDG	YDG	XP_029855797.1
Gymnogyps californianus	Cathartidae	Cathartiformes	FDG	YDG	XP_050767359.1
Indicator indicator	Indicatoridae	Piciformes	FDG	YDG	XP_054251821.1
Dryobates pubescens	Picidae	Piciformes	FDG	YDG	XP_054032364.1
**[Clade: Australaves]**					
Falco biarmicus	Falconidae	Falconiformes	FDG	YDG	XP_056217499.1
Falco peregrinus	Falconidae	Falconiformes	FDG	YDG	XP_005230374.2
Falco cherrug	Falconidae	Falconiformes	FDG	YDG	XP_005447158.2
Falco naumanni	Falconidae	Falconiformes	FDG	YDG	XP_040472898.1
Falco rusticolus	Falconidae	Falconiformes	FDG	YDG	XP_037266575.1
Strigops habroptila	Strigopidae	Psittaciformes	FDG	YDG	XP_030329392.1
Oenanthe melanoleuca	Muscicapidae	Passeriformes	LDG	YDG	XP_056367457.1
Agelaius phoeniceus	Icteridae	Passeriformes	LDG	YDG	XP_054504807.1
Molothrus ater	Icteridae	Passeriformes	LDG	YDG	XP_036254314.1
Melozone crissalis	Passerellidae	Passeriformes	LDG	YDG	XP_054149877.1
Vidua macroura	Viduidae	Passeriformes	LDG	YDG	XP_053854333.1
Vidua chalybeata	Viduidae	Passeriformes	LDG	YDG	XP_053819889.1
Serinus canaria	Fringillidae	Passeriformes	LDG	YDG	XP_030089066.1
Manacus candei	Pipridae	Passeriformes	LDG	YDG	XP_051664576.1
Pipra filicauda	Pipridae	Passeriformes	LDG	YDG	XP_027581588.1
Chiroxiphia lanceolata	Pipridae	Passeriformes	LDG	YDG	XP_032566731.1
Corapipo altera	Pipridae	Passeriformes	LDG	YDG	XP_027515670.1
Neopelma chrysocephalum	Pipridae	Passeriformes	LDG	YDG	XP_027551161.1
Lepidothrix coronata	Pipridae	Passeriformes	LDG	YDG	XP_017676965.1
Myiozetetes cayanensis	Tyrannidae	Passeriformes	LDG	YDG	XP_050182663.1
Corvus hawaiiensis	Corvidae	Passeriformes	LDG	YDG	XP_048184262.1
Corvus moneduloides	Corvidae	Passeriformes	LDG	YDG	XP_031989227.1
Corvus cornix cornix	Corvidae	Passeriformes	LDG	YDG	XP_039421372.1
Catharus ustulatus	Turdidae	Passeriformes	LDG	YDG	XP_032936008.1
Hirundo rustica	Hirundinidae	Passeriformes	LDG	YDG	XP_039941467.1
Parus major	Paridae	Passeriformes	LDG	YDG	XP_015506045.1
Pseudopodoces humilis	Paridae	Passeriformes	LDG	YDG	XP_005529344.1
Lonchura striata domestica	Estrildidae	Passeriformes	LDG	YDG	OWK52732.1
Taeniopygia guttata	Estrildidae	Passeriformes	LDG	YDG	XP_030117315.1
Camarhynchus parvulus	Thraupidae	Passeriformes	LDG	YDG	XP_030821556.1
Sturnus vulgaris	Sturnidae	Passeriformes	LDG	YDG	XP_014747840.1
Pyrgilauda ruficollis	Passeridae	Passeriformes	VDG	YDG	XP_041323353.1
Onychostruthus taczanowskii	Passeridae	Passeriformes	VDG	YDG	XP_041283034.1
Passer montanus	Passeridae	Passeriformes	VDG	YDG	XP_039551881.1
